# Small molecule-directed differentiation of submerged-cultured human nasal airway epithelia for respiratory disease modeling

**DOI:** 10.1016/j.xcrm.2026.102692

**Published:** 2026-03-23

**Authors:** Henriette H.M. Dreyer, Georgia-Nefeli Ithakisiou, Sacha Spelier, Malina K. Iwanski, Eugene Katrukha, Jonne Terstappen, Lisa W. Rodenburg, Aditi Shekhar, Loes A. den Hertog-Oosterhoff, Shannon M.A. Smits, Isabelle S. van der Windt, Lotte T. Azink, Linda H.M. Bijlard, Koen Passier, Sam F.B. van Beuningen, Robert Jan Lebbink, Eric G. Haarman, Cornelis K. van der Ent, Lukas C. Kapitein, Louis J. Bont, Jeffrey M. Beekman, Gimano D. Amatngalim

**Affiliations:** 1 Lab of Cellular Disease Models, Department of Pediatrics, Regenerative Medicine Center Utrecht, University Medical Center Utrecht, Utrecht, the Netherlands; 2 Cell Biology, Neurobiology and Biophysics, Department of Biology, Faculty of Science, Utrecht University, Utrecht, the Netherlands; 3 Center for Translational Immunology, University Medical Centre Utrecht, Utrecht, the Netherlands; 4 Department of Pediatrics, Wilhelmina Children’s Hospital, University Medical Centre Utrecht, Utrecht, the Netherlands; 5 Department of Medical Microbiology, University Medical Center Utrecht, Utrecht, the Netherlands; 6 Centre for Living Technologies, Eindhoven-Wageningen-Utrecht Alliance, Utrecht, the Netherlands; 7 Department of Paediatric Pulmonology, Emma Children’s Hospital, Amsterdam UMC, Amsterdam, the Netherlands; 8 Department of Pediatric Pulmonology, Wilhelmina Children’s Hospital, University Medical Centre Utrecht, Member of ERN-LUNG, Utrecht, the Netherlands

**Keywords:** airway epithelial cell cultures, submerged differentiation, nasal epithelial cells, mucociliary epithelium, patient-derived airway epithelial models, primary ciliary dyskinesia, cystic fibrosis, respiratory syncytial virus, RSV, airway organoids, high-throughput epithelial screening

## Abstract

Submerged cultures of undifferentiated or transformed epithelial cells are widely used in respiratory research due to their ease of use and scalability. However, these systems fail to capture the cellular diversity of the human airway epithelium. Here, we describe a submerged differentiation model using cryopreserved human nasal epithelial cells obtained via minimally invasive brushings. By targeting Notch and BMP signaling with small molecule inhibitors, we differentiate these cells into complex epithelial cultures containing basal, secretory, and ciliated cell types on standard plastic cultureware. This method supports scalable culture of both 2D epithelial monolayers and 3D organoids and is applied to disease modeling in primary ciliary dyskinesia, cystic fibrosis, and respiratory syncytial virus infection. The resulting system enables scalable assessment of disease-relevant epithelial functions in respiratory research.

## Highlights

• Notch and BMP inhibition enables mucociliary differentiation in submerged nasal epithelia
• Submerged-differentiated epithelial monolayers can be converted into 3D airway organoids
• S-diff cultures support functional disease modeling in PCD, CF, and RSV infection
• The model enables scalable epithelial phenotyping and therapeutic testing

## Graphical abstract

**Figure:**
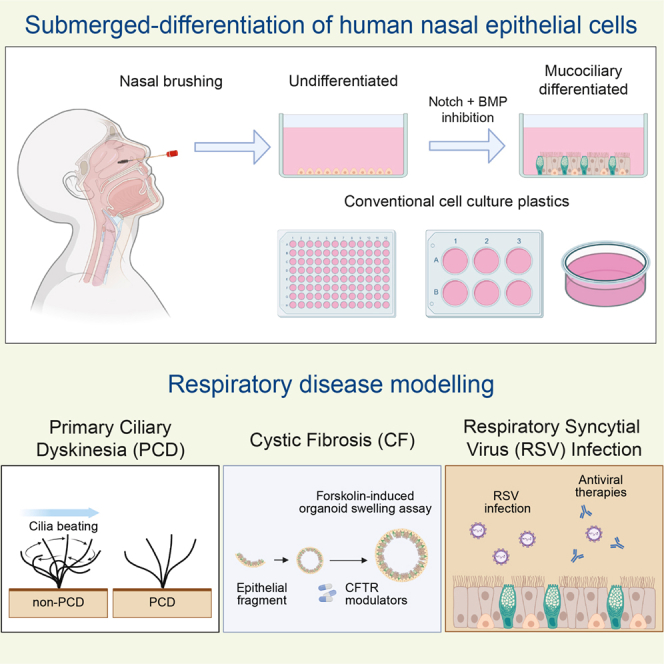

## Teaser

Dreyer et al. describe a submerged differentiation approach for human nasal epithelial cells that enables scalable generation of mucociliary epithelia and airway organoids. The model supports functional disease phenotyping and therapeutic testing in primary ciliary dyskinesia, cystic fibrosis, and respiratory syncytial virus infection.

## Introduction

Despite advancements in sophisticated epithelial models, such as transwell-differentiated epithelia, organoids, and organ-on-a-chip systems, cell cultures on conventional plastic substrates under fluid-submerged conditions remain a cornerstone of biomedical research and drug development.^1^ Their widespread use is due to their ease of handling, scalability, and cost-effectiveness, making them ideal for generating large datasets and performing drug screening assays. However, these traditional models often lack the cellular diversity of native epithelia, leading to suboptimal experimental outcomes and contributing to higher drug development failure rates.

This limitation is particularly pronounced in respiratory research, where accurate *in vitro* models of the human airway epithelium are essential for successful drug discovery.^2^ For nearly 40 years, the air-liquid interface (ALI) culture model has been the gold standard for studying airway epithelial cells.^3^ In this model, primary airway basal progenitor cells are differentiated into secretory and ciliated cells under air-exposed conditions, generating monolayers that closely resemble the native airway epithelium.^4^ While highly valuable for research and drug validation, application of ALI cultures is limited by the need for specialized transwell cultureware, which is costly and incompatible with certain assays. As a result, submerged cultures with undifferentiated airway basal cells (BCs) and epithelial cell lines (e.g., 16HBE, A549, Calu-3) remain commonly used, despite their inability to replicate the complex cellular composition of ALI cultures.^5^

Given the need for accessible and biologically relevant airway models, nasal cells have emerged as a convenient and reliable source of human airway epithelial cells for *in vitro* studies.^6^ Nasal cells are easily collected through minimally invasive nasal brushing and share functional characteristics with bronchial epithelia, making them a valuable surrogate in respiratory research.^7^ Due to these advantages, nasal cultures are increasingly used in personalized disease models, especially for monogenic diseases such as primary ciliary dyskinesia (PCD) and cystic fibrosis (CF).^6^^,^^8^ Additionally, nasal epithelial cells are widely applied to study respiratory virus infections, including respiratory syncytial virus (RSV).^9^^,^^10^

In this study, we aimed to bridge the gap between traditional submerged cultures and more advanced models by establishing a method in which cryopreserved human nasal airway epithelial cells are differentiated under submerged conditions on conventional culture plastics. By targeting Notch and BMP signaling pathways with small molecule inhibitors, we generated submerged cultures that reflect key aspects of the composition of human airway epithelium, containing a mix of basal, secretory, and ciliated cells. This method enables the generation of 2D epithelial monolayers in various plastic culture formats, as well as scalable production of 3D airway organoids. Furthermore, in line with previously published human airway organoid models,^11^^,^^12^ we applied submerged-differentiated human nasal epithelial cells (S-diff HNECs) to disease modeling in PCD, CF, and RSV infection. The resulting system supports scalable functional studies and medium-throughput applications, particularly for early-phase validation and functional testing across donor cohorts.

## Results

### Notch and BMP inhibition promotes the differentiation of submerged-cultured nasal epithelia

Hypoxic conditions in submerged cultures are associated with suppressed airway epithelial differentiation and enhanced activation of Notch and BMP signaling pathways.^13^^,^^14^^,^^15^^,^^16^ To promote differentiation in submerged nasal airway epithelial cultures, we, therefore, evaluated the effects of the Notch-targeting γ-secretase inhibitor DAPT and the BMP inhibitor DMH1, in combination with a previously established differentiation medium used for ALI cultures.^17^^,^^18^

**Figure 1:**
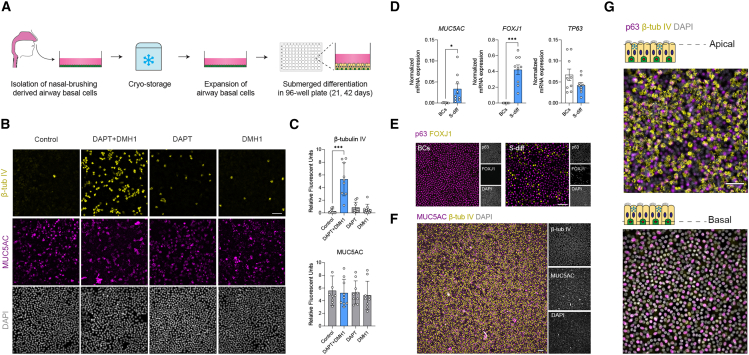
Submerged differentiation of human nasal epithelia with DAPT and DMH1 (A) Graphic illustration showing the workflow of expanding cryo-stored nasal brushing-derived human airway basal cells, followed by experiments investigating differentiation in submerged cultures. The control condition in this experiment refers to submerged cultures differentiated using the standard medium applied in ALI differentiation. (B) Representative immunofluorescent images of randomly selected S-diff HNECs after differentiation with DAPT and DMH1 for 21 days. Cells were fixed and stained for the ciliated cell marker β-tubulin IV (β-tub IV; yellow), secretory cell marker MUC5AC (purple), and DAPI (gray). (C) Quantification of β-tubulin IV (β-tub IV) and MUC5AC signal (*n* = 3 images for 3 independent donors). (D) Quantitative PCR comparing the expression of *MUC5AC*, *FOXJ1*, and *TP63* between BCs and S-diff HNECs of healthy donors (*n* = 2 replicates for 9 independent donors). mRNA expression was normalized to the average expression of housekeeping genes. (E) Representative immunofluorescent images of BCs and S-diff HNECs stained for the ciliated cell transcription factor FOXJ1 (yellow), basal cell transcription factor p63 (purple), and DAPI (gray). (F) Representative immunofluorescent images of S-diff HNECs differentiated for 42 days stained for β-tubulin IV (β-tub IV; yellow), MUC5AC (purple), and DAPI (gray). (G) Representative immunofluorescent images of S-diff HNECs differentiated for 42 days, demonstrating β-tubulin IV (β-tub IV; yellow) staining at the apical side (upper panel) and p63 staining (purple) located more at the basal side of the culture. Scale bars, 50 μm. Data are presented as mean ± SD, and individual data points. Statistical significance was tested using (A) two-way ANOVA with Dunnett’s multiple comparison test and (D) two-tailed paired *t* test. ∗*p* < 0.05 and ∗∗∗*p* < 0.001.

Submerged differentiation was assessed using cryopreserved human BCs derived from nasal brushings of healthy donors. These cells were cultured as confluent monolayers in conventional 96- well culture plates and differentiated for 21 or 42 days (Figure 1A). In contrast to human bronchial epithelial cells,^13^ Notch inhibition alone was insufficient to induce the differentiation of ciliated cells in nasal cultures. However, co-treatment with DAPT and DMH1 significantly increased the number of β-tubulin IV^+^ ciliated cells in S-diff HNECs compared to individual treatments (Figures 1B and 1C). The number of MUC5AC^+^ secretory cells remained consistent across conditions, indicating that secretory cell differentiation occurs effectively in submerged cultures even without DAPT or DMH1. This corresponded with a previous study, reporting persistent MUC5AC^+^ secretory cell differentiation in ALI-cultured HNECs under hypoxic conditions.^19^ DAPT combined with the BMP inhibitor Noggin also enhanced ciliated cell differentiation, whereas BMP4 co-stimulation inhibited this process (Figures S1A and S1B).

S-diff HNECs displayed significantly higher mRNA expression of *MUC5AC* and the ciliated cell-associated transcription factor *FOXJ1* compared to undifferentiated BCs, while expression of the BC transcription factor *TP63* remained unchanged (Figure 1D). Immunofluorescence staining confirmed these findings, showing that S-diff HNECs, but not BCs, contained both FOXJ1^+^ and p63^+^ nuclei (Figure 1E). In addition to MUC5AC, secretory epithelial markers SLPI, pIgR, and CC16 (SCGB1A1) were detected in S-diff HNECs (Figure S1C). S-diff HNECs maintained for up to 42 days in culture without passaging exhibited uniform cellular distribution within the epithelial monolayer (Figure 1F), with ciliated cells localized at the apical side and BCs at the basal side (Figure 1G), recapitulating the spatial organization of native airway epithelia. In summary, these results demonstrate that inhibition of Notch and BMP signaling effectively promotes the differentiation in submerged nasal airway epithelial cultures.

### Submerged-differentiated cultures display airway epithelial heterogeneity

**Figure 2:**
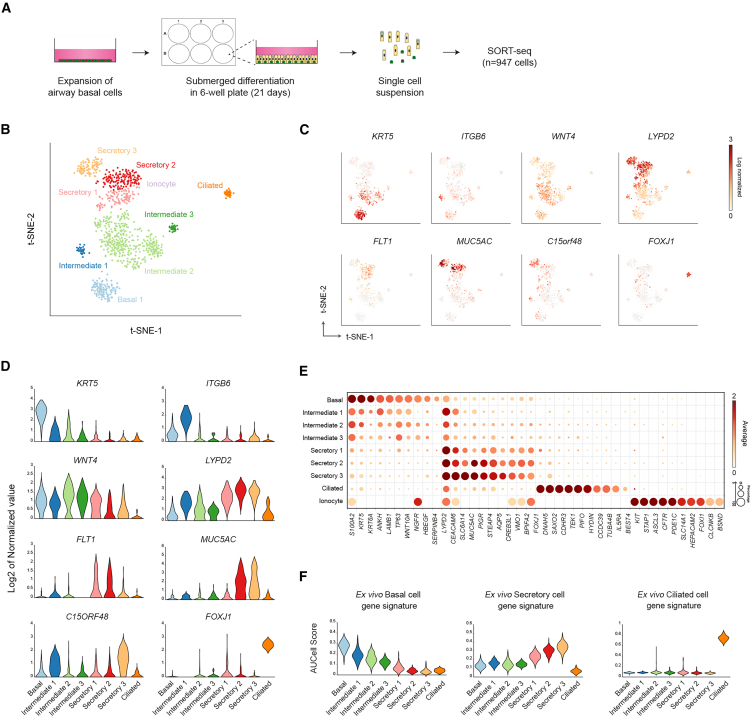
Single-cell RNA sequencing analysis of submerged-differentiated nasal epithelia (A) Graphic illustration showing the workflow of scRNA-seq experiments with S-diff HNECs. (B) t-SNE (t-distributed stochastic neighbor embedding) analysis of expression from scRNA-seq (947 cells in total) of HNECs (*n* = 3 independent donors), which were differentiated in submerged cultures for 21 days. (C) Clusters are labeled in t-SNE plots by cell identity based on marker gene expression. (D) Violin plots showing the expression of marker genes of identified cell subsets in scRNA-seq data. (E) Bubble heatmap showing the expression of selected marker gene expression of identified cell types in S-diff HNECs. (F) AUCell scores of *ex vivo* basal, secretory, and ciliated cell gene signatures in S-diff cultures.

To further characterize the cellular heterogeneity of S-diff HNECs, we performed single-cell RNA sequencing (scRNA-seq) using the SORT-seq (sorting and robot-assisted transcriptome sequencing) protocol,^20^ analyzing HNECs differentiated in 6-well plastic culture plates for 21 days (Figure 2A). We identified basal, intermediate, secretory, and ciliated cells (Table S1), which were further classified into nine distinct clusters representing airway epithelial subsets (Figure 2B). One basal cluster exhibited high expression of BC markers including *KRT5, TP63,* and *NGFR* (Figures 2C–2E). Intermediate subsets showed overlapping expression with basal and secretory groups, suggesting these populations represent transitional states during differentiation (Figures 2D and 2E), They were, furthermore, characterized by elevated *WNT4* expression (Figures 2C and 2D), previously implicated in promoting ciliated cell differentiation.^21^ Notably, we identified an intermediate subset marked by high *ITGB6* expression (Figures 2C and 2D), resembling basaloid-like cells described in ALI-differentiated cultures.^22^ Secretory cells, defined by high *LYPD2* expression and other secretory markers, were classified into three distinct subsets (Figures 2C–2E). This included one subset lacking *MUC5AC* but expressing VEGFR receptor 1 (*FLT1*) and two *MUC5AC*-expressing subsets (Figures 2C and 2D). One *MUC5AC*^+^ subset was marked by *C15ORF48* expression, a marker previously also identified in scRNA-seq analyses of native nasal airway epithelia.^23^ This subset displayed elevated expression of secretory markers, suggesting a more mature secretory cell phenotype compared to other subsets (Figure 2E). A distinct ciliated cell cluster was also identified based on high expression of *FOXJ1* and other cilia-related genes (Figures 2C–2E). Despite the presence of these major airway cell types, only a single ionocyte was detected (Figures 2B and 2E). To further validate cluster identities, we cross-referenced the gene expression of basal, secretory, and ciliated cell gene signatures derived from *ex vivo* nasal epithelium in the integrated Human Lung Atlas^24^ (Figure 2F). AUCell scores confirmed enrichment of the expected subsets in S-diff HNECs. Furthermore, gene signature scoring revealed a progressive decline in basal identity and an increase in secretory signatures across basal and intermediate clusters. Together, these results demonstrate that S-diff HNEC cultures contain major airway epithelial cell types and exhibit cellular heterogeneity.

### Comparable transcriptomes between S-diff and ALI-differentiated HNECs

**Figure 3:**
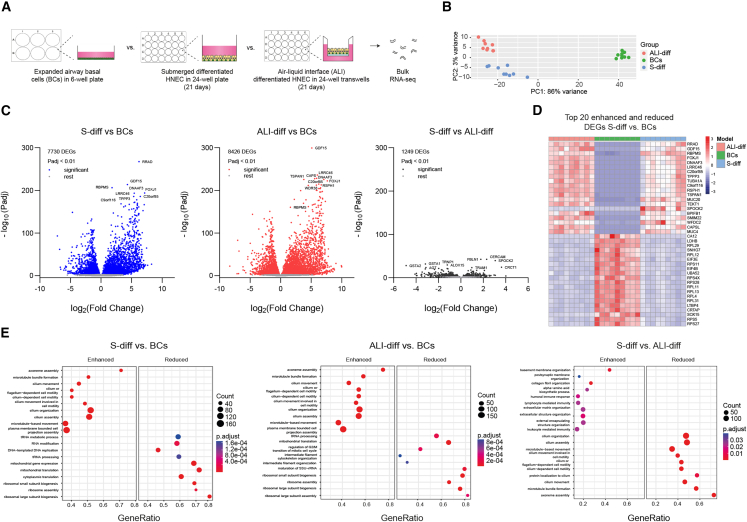
Comparative analysis of S-diff HNECs with BCs and ALI-differentiated HNECs (A) Graphic illustration showing the use of expanded airway basal cells and S-diff and ALI-differentiated HNECs for RNA sequencing (RNA-seq) (*n* = 9 independent donors). (B) PCA plot from bulk RNA-seq data, comparing S-diff HNECs with BCs and ALI-differentiated cultures. (C) Volcano plots displaying the log_2_ fold change and the log_10_ DEGs in S-diff HNECs compared to BCs (left), ALI-differentiated cultures compared to BCs (middle), and S-diff HNECs compared to ALI-differentiated cultures (right). For all three comparisons, the top 10 DEGs are mentioned by name. (D) Heatmap showing marker gene expression of top 20 enhanced and reduced DEGs of S-diff HNECs vs. BCs. (E) GO term analysis of the top 10 activated and suppressed biological processes in S-diff vs. BCs (left), ALI-differentiated vs. BCs (middle), and S-diff vs. ALI-differentiated HNEC (right).

To assess transcriptomic similarities between S-diff HNECs, undifferentiated BCs, and ALI-differentiated cultures, we performed bulk RNA sequencing (Figure 3A). The differentiation medium used for both S-diff and ALI cultures included DAPT and DMH1, with ALI conditions yielding a higher proportion of ciliated cells (Figure S2A).

Principal-component analysis (PCA) showed that S-diff HNECs cluster closer to ALI-differentiated cells than to BCs, with 3% and 86% of variance across PC1 and PC2, respectively (Figure 3B). Differential gene expression analysis revealed 7,730 differentially expressed genes (DEGs) between S-diff-HNECs and BCs, 8,426 DEGs between ALI-diff-HNECs and BCs, and 1,249 DEGs between S-diff-HNECs and ALI cultures (*p* < 0.01) (Figure 3C; Table S2). The DEG overlap between S-diff vs. BC and ALI vs. BC comparisons (Figures 3D and S2B) suggests a shared differentiation trajectory. Analysis of epithelial subset markers showed reduced BC-associated gene expression and elevated secretory and ciliated cell marker expression in both S-diff and ALI cultures relative to BCs (Figure S2C). However, S-diff HNECs displayed significantly lower expression of ciliated-cell markers compared to ALI, in line with the lower proportion of ciliated cells. Gene Ontology (GO) term analysis indicated shared enrichment of gene sets related to cilia structure and motility in both S-diff and ALI cultures and a downregulation of transcription- and translation-associated pathways compared to BCs. DEGs between S-diff and ALI cultures were enriched for pathways linked to ciliary function, extracellular matrix (ECM) organization, and immune signaling (Figures 3E and S2D). For example, genes elevated in S-diff cultures included *CERCAM*, *FBLN1*, and *SPOCK2* (ECM remodeling) as well as *IL1R1*, *IL20RB*, and *FSTL1* (immune signaling). However, the functional significance of many DEGs (e.g., *TRAM1*, *CRCT1*, and *LGALS3BP*) remains unclear, as these are not well studied in airway epithelial biology. To investigate whether extended culture time enhances maturation, we differentiated S-diff HNECs for up to 42 days (Figure S3A). FOXJ1 mRNA expression and β-tubulin IV staining were highest at day 42, alongside reduced MUC5AC expression, suggesting ongoing *trans*-differentiation from secretory to ciliated phenotypes (Figures S3B–S3D). Expression of the ionocyte-associated transcription factor *FOXI1* and *CFTR* also were highest at day 42, further supporting progressive epithelial maturation (Figure S3B). Altogether, bulk RNA-seq data suggest that S-diff HNECs are more comparable to ALI-differentiated HNECs when compared to undifferentiated BCs. Extending the differentiation period in S-diff cultures increases the number of ciliated cells, further aligning their cellular composition with ALI-differentiated cultures.

### Cilia dynamics of submerged-differentiated cultures of healthy controls and PCD subjects

As a first showcase demonstrating the suitability of S-diff HNECs for studying respiratory diseases, we investigated its potential in the context of PCD. This heterogeneous monogenic disorder is characterized by airway epithelial ciliopathy, with mutations reported in approximately 50 different PCD-related genes.^8^ These mutations can lead to cilia immotility or abnormal wave patterns in motile cilia, reduced assembly of cilia structures, or attenuated ciliated cell differentiation.

**Figure 4:**
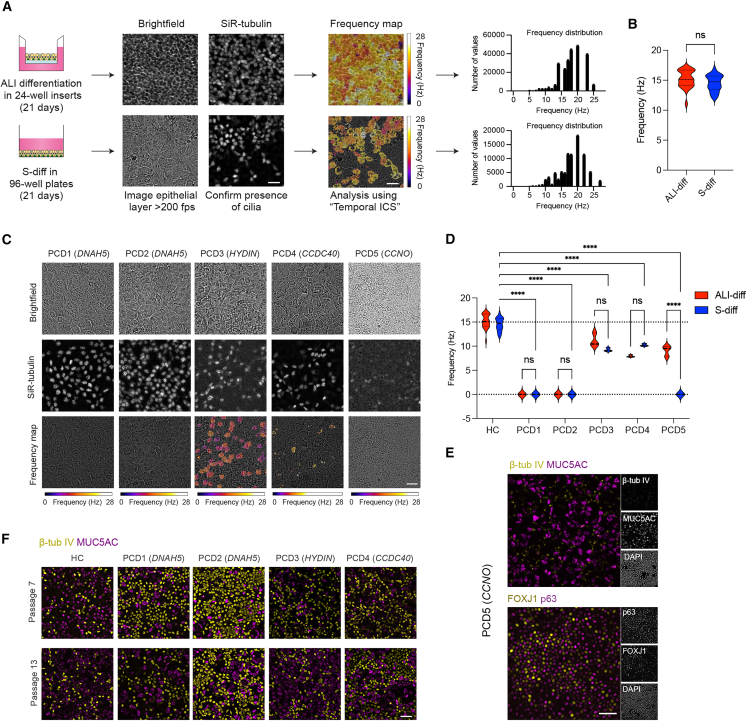
CBF in S-diff HNECs of healthy controls and PCD subjects (A) Graphic illustration showing the workflow of CBF measurements. Representative bright-field and immunofluorescent video frames of ALI- and S-diff HNECs incubated with the live dye SiR-tubulin with corresponding frequency map generated with “Temporal ICS” analysis. Video frames were extracted from Videos S1 and S2. Frequency maps show CBF in hertz (Hz). Color bar indicates CBF from 0 to 28 Hz. (B) Average CBF in Hz of submerged and ALI-differentiated cultures (*n* = 3 videos for 9 independent healthy donors). Data are shown as violin plots. (C) Representative bright-field and immunofluorescent video frames of submerged-differentiated PCD cultures (PCD1–5) incubated with the live dye SiR-tubulin with corresponding frequency maps and measured CBF. Frequency is presented in Hz. Color bar indicates CBF from 0 to 28 Hz. (D) CBF in Hz of ALI- and submerged-differentiated healthy (*n* = 9) and PCD (*n* = 5, PCD1-5) donors, shown as violin plot; dotted lines indicate average CBF of healthy subject cultures. (E) Representative immunofluorescent image of S-diff HNECs of a PCD donor with mutations in CCNO (PCD5) stained for β-tubulin IV (β-tub IV; yellow), MUC5AC (purple) (top), p63 (purple), and FOXJ1 (yellow) (bottom), and DAPI (gray). (F) Representative immunofluorescent images of p7 and p13 S-diff HNECs of one healthy donor and PCD subjects PCD1–4. Cells were fixed and stained for the secretory cell marker MUC5AC (purple) and ciliated cell marker β-tubulin IV (yellow). Scale bars, 50 μm. Statistical significance was tested using (B) a two-tailed paired *t* test and (D) a two-way ANOVA with Dunnett’s multiple comparison. ns, non-significant; ∗∗∗∗*p* < 0.0001.

First, we compared cilia motility in S-diff cultures (Video S1) to ALI cultures (Video S2) of healthy controls (HCs) by measuring the ciliary beat frequency (CBF), which did not differ significantly (Figures 4A and 4B). Monitoring cilia motility during the differentiation of submerged cultures demonstrated a stable CBF between 21 and 42 days (Figure S4A). We next assessed ciliary function in HNECs from PCD subjects with distinct genotypes. In line with previous studies,^25^^,^^26^ we did not detect ciliary movement in PCD subjects with *DNAH5* mutations in either S-diff or ALI cultures (Figures 4C, 4D, and S5; Videos S3 and S4). Cultures from a subject with *HYDIN* mutations showed significantly reduced CBF in both models (Video S5), while *CCDC40*-mutant cultures displayed partially motile cilia with lower CBF compared to HCs (Video S6). These findings were consistent across S-diff and ALI conditions. In contrast, for the donor carrying *CCNO* mutations, both models showed impaired cilia formation with only occasional single cilia detectable by microscopy (Figures 4E and S5A). However, ciliary motion was observed exclusively in ALI cultures. In S-diff cultures from the same donor, FOXJ1 protein was abundantly expressed (Figure 4E).

Video S1. Cilia motility in submerged-differentiated human nasal epithelial cells of a healthy control

Video S2. Cilia motility in air-liquid interface differentiated human nasal epithelial cells of a healthy control

Video S3. Cilia motility in submerged-differentiated human nasal epithelial cells of a PCD subject with *DNAH5* mutations

Video S4. Cilia motility in air-liquid interface-differentiated human nasal epithelial cells of a PCD subject with *DNAH5* mutations

Video S5. Cilia motility in submerged-differentiated human nasal epithelial cells of a PCD subject with *HYDIN* mutations

Video S6. Cilia motility in submerged-differentiated human nasal epithelial cells of a PCD subject with *CCDC40* mutations

To validate prolonged use of S-diff HNECs in personalized screening assays, we examined BCs from HC and PCD donors and expanded up to passages 6 and 12 in feeder-free expansion conditions (Figure S4B). We observed consistent population doublings and the absence of morphological differences until passage 12 in all examined donors (Figures S4C and S4D). MUC5AC and β-tubulin IV staining of S-diff HNECs were similar between passages 7 and 13 in HC donors; however, significant differences were observed in PCD donors (Figures 4F and S4E). Despite this variability, the average CBF of S-diff HNECs at passages 7 and 13 were comparable for both HC and PCD donors (Figure S4F) and corresponded with observations at passage 4 (Figure 4D). To explore potential therapeutic applications of the S-diff model in PCD, we tested the efficacy of the readthrough compounds G418 and ELX-02^27^ in S-diff cultures from PCD donors carrying premature termination codon (PTC) mutations in *DNAH5* and *CCDC40* (Figure S6; Table S3). No functional improvement in CBF was observed following treatment. This is consistent with previous studies using ALI cultures, which also failed to demonstrate cilia rescue in PCD patients with nonsense mutations in *MCIDAS*.^28^

Collectively, we demonstrate that CBF measurements in S-diff HNECs remain stable over extended passages, mirror observations in ALI cultures, and reflect genotype-specific ciliopathy phenotypes in PCD. This supports the application of the S-diff model for both phenotyping and early-phase drug testing.

### CFTR modulator responses in CF nasal airway organoids generated from S-diff monolayers

Next, we investigated the application of S-diff HNECs as a model for CF airway disease. CF is caused by autosomal recessive mutations in the CF transmembrane conductance regulator (CFTR) gene, leading to defective CFTR protein function in airway epithelial cells. This results in impaired anion and fluid secretion, dehydration of secreted mucins, and defective mucociliary clearance due to mucus stasis.^29^ CFTR-modulating drugs, i.e., correctors and potentiators, can restore CFTR function,^30^ but treatment efficacy is highly genotype dependent.

**Figure 5:**
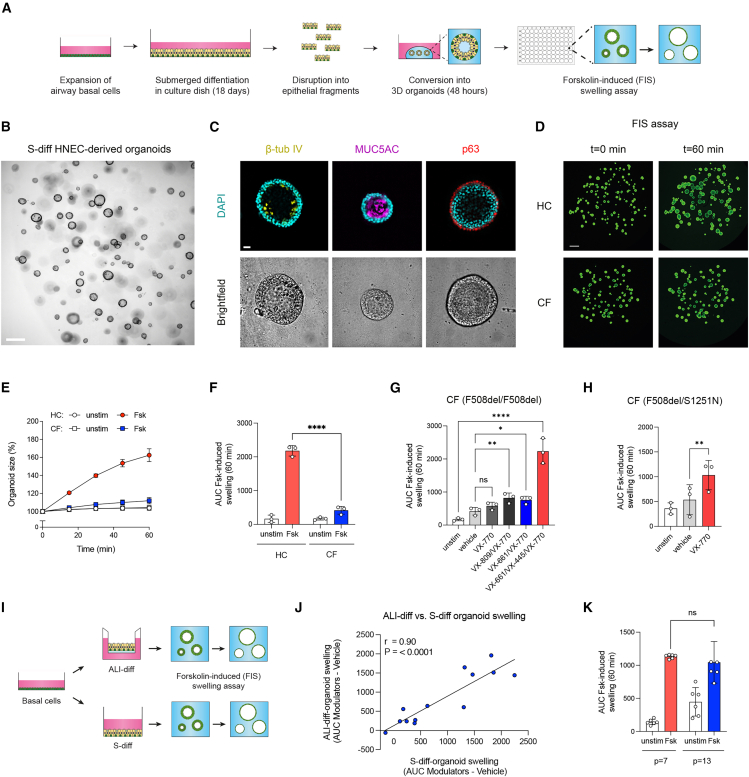
Conversion of S-diff HNECs into 3D organoids and CFTR function measurements (A) Graphic illustration showing the workflow of generating airway organoids from epithelial fragments of S-diff HNECs, and application in forskolin-induced swelling assays. (B) Representative bright-field images of cystic airway organoids formed after 48 h from S-diff monolayer cultures. Scale bar, 250 μm. (C) Immunofluorescent (top) and bright-field (bottom) images of S-diff HNEC-derived organoids of β-tubulin IV (yellow, left top), MUC5AC (purple, middle top), p63 (red, right top), and DAPI (cyan). Scale bar, 10 μm. (D) Representative images of forskolin-induced swelling determined with calcein green AM ester-stained HC and CF (F508del/F508del) organoids, with images taken before stimulation with forskolin (t = 0, left) and 60 min after stimulation (t = 60 min, right). Scale bar, 500 μm. (E and F) Comparison of FIS between HC and CF airway organoids derived from S-diff HNECs (both *n* = 3 independent donors) that were unstimulated or stimulated with forskolin (Fsk). (G) CF F508del homozygous organoids derived from S-diff HNECs (*n* = 3 independent donors) were pre-treated with vehicle or CFTR correctors, VX-809, or VX-661/VX-445 for 48 h. Afterward, FIS was determined following acute stimulation with Fsk, VX-770, or vehicle. (H) FIS responses in CF F508del/S1251N organoids (*n* = 3 donors) following acute stimulation with VX-770. (I) Schematic overview of the comparison between FIS responses in airway organoids derived from ALI- vs. S-diff cultures. (J) Correlation plot of FIS responses between ALI-derived and S-diff-derived organoids from the same CF donors (Pearson r = 0.90, 95% confidence interval, 0.70–0.97; *p* < 0.0001). (K) FIS in organoids generated from HC basal cells expanded to passage 3 or 7 before S-diff and organoid formation (*n* = 2 donors). FIS results are depicted as (E) the percentage change in surface area relative to t = 0 (normalized area) measured at 15-min time intervals for 60 min or (F–H and K) as area under the curve (AUC) plots (t = 60 min). Data are presented as mean ± SD and individual data point. Statistical significance was tested using a (F and I) two-way ANOVA with Dunnett’s multiple comparison test, (G and H) Tukey’s multiple comparison test, (J) Pearson correlation analysis. ns, non-significant, ∗*p* < 0.05, ∗∗*p* < 0.01, ∗∗∗∗*p* < 0.0001.

We previously established a method to generate airway organoids from ALI-differentiated epithelial fragments for CFTR modulator screening using the forskolin-induced swelling (FIS) assay.^17^^,^^18^ Here, we tested whether S-diff HNEC cultures grown in scalable thermosensitive dishes could similarly be converted into organoids and applied in FIS-based drug testing (Figure 5A). At day 18 of differentiation—selected based on abundant secretory cell presence, the main CFTR-expressing population—epithelial monolayers were dissociated by cold-induced detachment. When embedded in ECM, the resulting fragments self-organized into cystic organoids within 48 h (Figure 5B). Immunofluorescence staining for β-tubulin IV, MUC5AC, and p63 confirmed the presence of ciliated, secretory, and BC types (Figure 5C). Because CFTR expression is relatively low in day 18 S-diff cultures, we performed FIS assays under inflammatory conditions that we previously showed to enhance CFTR expression and function in ALI-derived airway organoids.^17^ Under these conditions, CF organoids showed significantly reduced swelling compared to HCs, consistent with impaired CFTR function (Figures 5D–5F). In F508del/F508del CF organoids, swelling was restored upon treatment with the modulator combinations VX-809/VX-770, VX-661/VX-770, and VX-661/VX-445/VX-770 (Figure 5G). The CFTR potentiator VX-770 also increased swelling in organoids from individuals with an S1251N gating mutation (Figure 5H).

FIS responses in S-diff-derived organoids correlated strongly with those in ALI-derived organoids from the same donors (Pearson r = 0.90, *p* < 0.0001), indicating high concordance between models (Figures 5I, 5J, and S7). Organoids generated from higher-passage BCs (passages 7 and 13) from HC donors showed reduced swelling compared to passage 4, but similar responses to each other (Figure 5K), suggesting that CFTR function declines with increasing passage but stabilizes thereafter.

Altogether, these results demonstrate that S-diff monolayers can be efficiently converted into airway organoids suitable for CFTR function testing. This supports the use of the S-diff platform as a scalable and genotype-specific disease model for CF.

### RSV infections in submerged-differentiated airway epithelial cells

**Figure 6:**
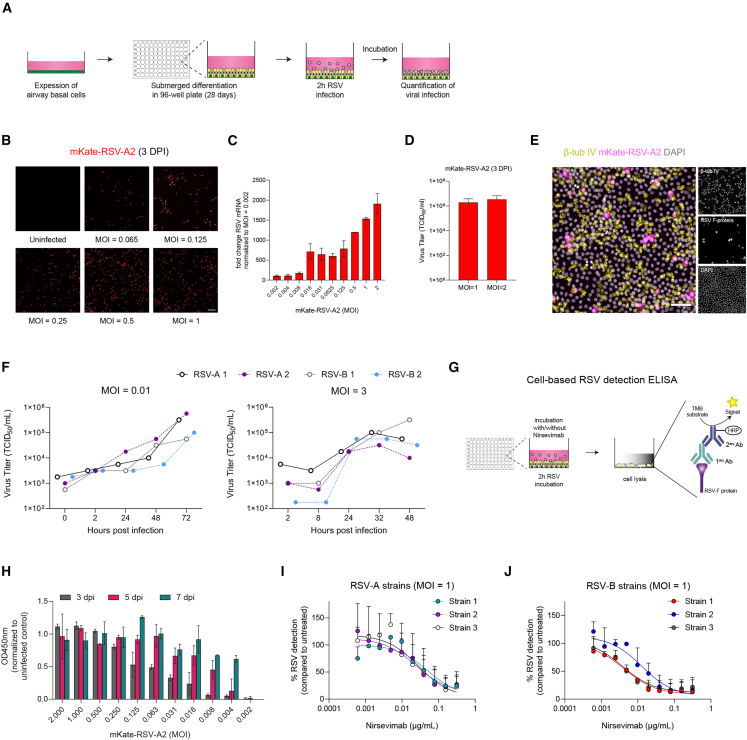
Modeling respiratory syncytial virus infections in S-diff HNECs (A) Graphic illustration summarizing experimental workflow of RSV infections in 96-well plate cultures S-diff HNECs. (B) Live imaging of RSV-A2 mKate (red)-infected S-diff HNECs underlines an MOI-dependent infection at 3 DPI. (C) RSV mRNA levels in mKate-RSV-A2-infected S-diff HNECs at 3 DPI. Data are shown as fold change, normalized to the lowest MOI (0.002) and housekeeping genes. (D) Infectious viral titers reported as TCID50 per mL for RSV-A2 mKate using a TCID50 assay. S-diff HNECs were infected with RSV-A2 mKate at MOI = 2 and MOI = 1, and subsequently, viral supernatants were collected and tittered with a TCID50 assay at 3 DPI. (E) Representative immunofluorescent images of S-diff HNECs infected with mKate-RSV-A2 (magenta) of β-tubulin IV (yellow) and DAPI (cyan). (F) S-diff HNECs were infected with clinical RSV-A and -B strains at an MOI = 0.01 (left) or MOI = 3 (right). Viral growth kinetics was determined by titrating supernatant and cell-bound RSV at different time intervals following infection. (G) Graphic illustration describing the concept of the cell-based ELISA used with S-diff HNECs to quantify RSV infections. (H) S-diff HNECs were infected with RSV-A2 mKate at an MOI range, and RSV infectivity was measured and depicted as OD450 nm corrected to uninfected control after 3, 5, and 7 DPI. (I and J) Assessment of the neutralization activity of Nirsevimab against clinical (I) RSV-A and (J) RSV-B strains. RSV infections in S-diff HNECs were conducted with MOI = 1 at 3 DPI. RSV infections were quantified by ELISA and depicted as the percentage RSV detection compared to uninfected controls. Scale bars, 50 μm. Experiment were conducted 2–3 times in S-diff HNECs of one healthy donor. Data are presented as mean ± SD.

Next, we used S-diff HNECs to study infections with RSV (Figure 6A), which imposes a significant health burden on vulnerable populations such as infants in low- to middle-income countries and individuals with chronic respiratory diseases.^9^

First, we infected S-diff cultures with an mKate-RSV-A2 strain, observing an MOI-dependent increase in mKate-fluorescence at 3 days post-infection (DPI) (Figure 6B). qPCR analysis confirmed RSV mRNA expression in cell lysates (Figure 6C), and titration of clarified cell pellets on HEp-2 cells demonstrated the production of infectious virions (Figure 6D). mKate-RSV-A2 fluorescence co-localized in β-tubulin IV^+^ cells (Figure 6E), consistent with tropism for ciliated cells. Comparative studies demonstrated higher RSV infection in the ALI model (Figures S8A and S8B), in line with a higher abundance of ciliated cells. Further infection studies were conducted with RSV-A and -B clinical-isolate strains demonstrating a time-dependent increase in the production of infectious virions in both S-diff HNECs and HEp-2 cells (Figures 6F and S8C). Furthermore, under a high viral infection condition (MOI = 3), a plateau in production of infectious virions was reached, indicating saturation of infectivity.

Matching the scalable character of the S-diff culture model, we optimized a cell-based ELISA, to simplify and increase the throughput of RSV infection measurements (Figure 6G). With the ELISA, we observed MOI-dependent RSV detection after infection with mKate-RSV-A2 in S-diff HNECs at 3 DPI (Figure 6H). Furthermore, at MOIs ranging from 0.004 to 0.125, a time-dependent increase in RSV detection was observed, while at higher MOIs, RSV detection was already saturated at 3 DPI. Validation of the ELISA demonstrated adequate assay performance (coefficient of variation values: 12.7% and 9.4%, respectively) and the ability to discriminate noise and signal (Z′-factor of 0.48) (Figure S5D). Moreover, we observed a positive correlation (rs2 = 0.73, *p* < 0.005) with the qPCR-based quantification of RSV F-mRNA in S-diff HNECs (Figure S8E). With the ELISA, we observed considerably lower RSV infections in S-diff HNECs than in HEp-2 and VERO E6 cells, confirming previous studies describing higher levels of infection in transformed cell lines^31^ (Figure S8F).

Last, we confirm the application of the cell-based ELISA with S-diff HNECs in viral neutralization experiments with the RSV-neutralizing monoclonal antibody nirsevimab.^32^ Here, we observed a dose-dependent decrease in the detection of RSV in infection experiments with mKate-RSV-A2, RSV-A, and RSV-B clinical-isolate strains (Figures 6I, 6J, and S8G). Consistent with previous studies in other model systems,^32^ we, furthermore, observed a near-complete inhibition at saturating concentration and an IC50 of 4.28 ng/mL. In summary, we demonstrate the feasibility of using S-diff HNECs in RSV infections and viral neutralization experiment together with a cell-based RSV detection ELISA.

## Discussion

In this study, we established a scalable method for small molecule-directed differentiation of submerged-cultured human nasal epithelial cells and examined its application for respiratory disease modeling. While previous studies have explored submerged differentiation of bronchial and murine airway cells,^13^^,^^33^ our approach builds on prior work in three ways. First, we use cryopreserved nasal cells obtained via non-invasive brushings, a clinically accessible, donor-specific source increasingly used in personalized respiratory research. Second, we show that a combination of DAPT and DMH1 enables differentiation into complex epithelial cultures containing basal, secretory, and ciliated cells under standard submerged conditions. Third, we demonstrate experimental applications of this model in PCD, CF, and RSV infection. Together, these features support the use of S-diff cultures as an accessible and scalable epithelial system, bridging the gap between traditional submerged systems and advanced but less-practical models such as ALI cultures, airway-on-chip systems, and *ex vivo* explants (Figure S6A).

One practical feature of the S-diff model is the simplicity of using conventional culture plastics and submerged conditions, which reduces cost and technical barriers. In addition, S-diff monolayers from large plastic surfaces can be used to generate 3D organoids, reducing the use of basement membrane extract normally required for organoid expansion and improving the efficiency of culturing uniformly differentiated airway organoids at scale. Comparative estimates of cost and labor (Figure S6B) confirm that while immortalized lines remain cheaper, they lack epithelial complexity and donor specificity. Conversely, ALI cultures are more complex but less scalable. The S-diff model occupies an intermediate position, combining scalability with disease relevance.

To explore its translational potential, we tested the S-diff model across three representative airway diseases. First, for PCD, head-to-head analysis of CBF measurements revealed consistent genotype-specific abnormalities in both models, with subtle differences in ciliary motility in specific cases (e.g., CCNO). In addition, readthrough compound testing in S-diff cultures from PCD donors carrying PTC mutations showed no improvement in ciliary motility, results consistent with earlier findings in ALI cultures.^28^ In CF, airway organoids derived from S-diff and ALI-diff monolayers displayed highly concordant CFTR modulator responses, with strong correlation in FIS measurements across genotypes. For RSV infection, we developed a scalable ELISA assay for virus detection in 96-well format, suitable for screening clinical isolates and antiviral compounds. Using this assay, we validated the infectivity of clinical RSV-A and -B, and assessed antiviral responses with nirsevimab. Compared with conventional epithelial cell lines, S-diff cultures provided a more physiologically relevant context for infection and antiviral testing. Together, these findings show that S-diff cultures can be used across multiple respiratory disease settings, for phenotyping, therapeutic screening, and evaluation of antiviral responses, while maintaining scalability and donor specificity. For both PCD and CF, S-diff cultures can be applied in diagnostic and donor-specific drug testing studies, including readthrough compounds or gene/mRNA-based therapies,^34^^,^^35^ while for RSV, they provide a platform to assess novel antiviral strategies. Moreover, this system may facilitate studies on host-virus interactions in individuals with immune deficiencies, as shown for a CD14-deficient patient using ALI cultures.^10^

While the S-diff model enables scalable differentiation of donor-derived nasal epithelial cells, several limitations remain. Our characterization primarily focused on ciliated and secretory lineages, and additional work is needed to define rare epithelial subsets. Ionocytes, for example, were not reliably detected, despite identification of a single ionocyte in scRNA-seq. Including additional small molecules, for instance targeting Sonic Hedgehog, may enhance differentiation efficiency or selectively promote these rare populations.^36^ Although transcriptomic profiles of S-diff cultures more closely resembled ALI cultures than BCs, they showed reduced ciliation and altered immune and ECM signaling. The functional implications of these differences remain to be defined. The model’s applicability is also context dependent. The lack of apical-basolateral polarity limits use in barrier function or aerosol exposure studies. However, for phenotypic screening, host-pathogen interaction studies, and donor-stratified disease modeling, S-diff cultures offer key advantages over immortalized cell lines. Finally, while day 42 emerged as a robust endpoint for mature ciliated differentiation, earlier time points (e.g., day 18–21) may be more suitable for secretory-cell-focused application, highlighting the need to match timing with functional assay design.

In conclusion, we describe submerged-differentiated nasal epithelia as a model for respiratory disease research, combining the simplicity of traditional cell cultures with the cellular complexity of the human airway epithelium. Similar to human airway organoids,^11^^,^^12^ we demonstrate proof-of-concept applications in PCD, CF, and RSV infection. This system supports the development of cost-efficient, high-content, and scalable epithelial models, enabling early-phase therapeutic screening, disease phenotyping, and donor-specific drug testing. Moreover, the differentiation strategy described here may be adapted to other epithelial tissues, broadening its relevance for translational and disease-focused research.

### Limitations of the study

This study has several limitations. Although submerged-differentiated cultures display major nasal epithelial cell types, rare populations such as ionocytes were not robustly detected. Further optimization, for example, through alternative small-molecule modulation, may be required to enhance epithelial diversity. In addition, S-diff cultures lack apical-basolateral polarity, limiting their use in applications that require directional transport, barrier measurements, or aerosol exposure; accordingly, this model is intended to complement rather than replace ALI cultures, chip-based systems, or *ex vivo* models. While direct comparisons with ALI cultures were performed for ciliary function in PCD and CFTR modulator responses in CF, broader validation across additional disease contexts and perturbations will be needed to define the predictive range of the model. Functional outcomes depend on differentiation stage, and optimal time points may vary by application, underscoring the need to align culture timing with the specific experimental question. Finally, due to limited sample sizes within donor groups, the study was not powered to detect sex- or age-specific effects, and, therefore, no stratified analyses were performed.

## Resource availability

### Lead contact

Further information and requests for resources and reagents should be directed to and will be fulfilled by the lead contact, Dr. Gimano D. Amatngalim (g.d.amatngalim@umcutrecht.nl).

### Materials availability

This study did not generate new unique reagents.

### Data and code availability

• Bulk and single-cell RNA-seq data generated in this study have been deposited in the NCBI Gene Expression Omnibus (GEO) database under accession numbers GEO: GSE318346, and GSE318347. De-identified raw count matrices are publicly available through this repository. Raw sequencing files are not publicly available due to privacy restrictions related to human subjects. To request access to the raw sequencing files, please contact the lead contact.
• No custom code was generated in this study.
• Any additional information required to reanalyze the data reported in this work paper is available from the lead contact upon request.

## Acknowledgments

This study was supported by grants of 10.13039/100016036Health∼Holland Top Consortium Knowledge and Innovation (TKI) (LSHM18062), ZonMw (114025009), the Dutch Cystic Fibrosis Foundation (NCFS, HIT-CF grant), and the Netherlands Organization for Scientific Research (NWO) Gravitation program IMAGINE! (project number 24.005.009) and by funding from AstraZeneca. This study is part of the Cystic Fibrosis Transition Project of the Ombion Center for Animal-free Biomedical Translation program in the Netherlands that is financed by a National Growth Fund (NGFCPBT241).

## Author contributions

Conception and/or design, H.H.M.D., G.-N.I., S.S., L.J.B., J.M.B., and G.D.A.; acquisition, analysis, or interpretation of data, H.H.M.D., G.-N.I., S.S., M.K.I., E.K., J.T., L.W.R., A.S., L.A.d.H.-O., S.M.A.S., I.S.v.d.W., L.T.A., L.B., K.P., S.F.B.v.B., R.J.L., E.G.H., C.K.v.d.E., L.C.K., J.M.B., and G.D.A.; drafting the work or revising it critically for important intellectual content, H.H.M.D., G.-N.I., S.S., L.H.M.B., J.B., and G.A.; final approval of the version submitted for publication, H.H.M.D., G.I., S.S., M.K.I., E.K., J.T., L.W.R., A.S., L.A.d.H.-O., S.M.A.S., I.S.v.d.W., L.T.A., L.H.M.B., K.P., S.F.B.v.B., R.J.L. E.G.H., C.K.v.d.E., L.C.K., J.M.B., and G.D.A.

## Declaration of interests

J.M.B. has regular interaction with pharmaceutical and other industrial partners and received nonfinancial support from Vertex Pharmaceuticals and personal fees and nonfinancial support from Proteostasis Therapeutics, outside the submitted work. J.M.B. reports grants from Galapagos NV, Proteostasis Therapeutics, and Eloxx Pharmaceuticals, outside the submitted work. J.M.B. has a patent granted (20210333266) related to CFTR function measurements in organoids and received personal fees from HUB/Royal Dutch Academy of Sciences, during the conduct of the study. He co-founded FAIR therapeutics BV and has a minority shareholders position. C.K.v.d.E. reports grants from GSK, Nutricia, TEVA, Gilead, Vertex, ProQR, Proteostasis, Galapagos NV, Eloxx, and Santhera, all paid to UMCU; C.K.v.d.E. has a patent granted (20210333266) related to CFTR function measurements in organoids and received personal fees from HUB/Royal Dutch Academy of Sciences, during the conduct of the study. L.J.B. has regular interaction with pharmaceutical and other industrial partners. He has not received personal fees or other personal benefits. His institution, University Medical Center Utrecht (UMCU), has received major funding (>€100,000 per industrial partner) from AbbVie, MedImmune, AstraZeneca, Sanofi, Janssen, Pfizer, MSD, and MeMed Diagnostics. UMCU has received major funding for the RSV GOLD study from the Bill and Melinda Gates Foundation. UMCU has received major funding as part of the public private partnership IMI-funded RESCEU and PROMISE projects with partners GSK, Novavax, Janssen, AstraZeneca, Pfizer, and Sanofi. UMCU has received major funding by Julius Clinical for participating in clinical studies sponsored by MedImmune and Pfizer. UMCU received minor funding (€1,000–25,000 per industrial partner) for consultation and invited lectures by AbbVie, MedImmune, Ablynx, Bavaria Nordic, MabXience, GSK, Novavax, Pfizer, Moderna, AstraZeneca, MSD, and Sanofi, Janssen. L.J.B. is the founding chairman of the ReSViNET Foundation.

## Declaration of generative AI and AI-assisted technologies in the writing process

During the preparation of this work, the authors used ChatGPT 4.0 in order to improve language and readability. After using this tool, the authors reviewed and edited the content as needed and take full responsibility for the content of the publication.

## STAR★Methods

### Key resources table

**Table:** 

REAGENT or RESOURCE	SOURCE	IDENTIFIER
**Antibodies**		

Anti-beta IV Tubulin antibody [EPR16776]	Abcam	Cat#ab179509; RRID: AB_2716759
Anti-Beta-Tubulin IV [ONS1A6]	Emergo Biogenex	Cat#MU178-UC; RRID: AB_2335625
MUC5AC Monoclonal Antibody (45M)	Thermo Fisher Scientific	Cat#MA1-38223; RRID: AB_2266697
Anti-p63 antibody [EPR5701]	Abcam	Cat#ab124762; RRID: AB_10971840
FOXJ1 Monoclonal Antibody (2A5)	Thermo Fisher Scientific	Cat#14-9965-80; RRID: AB_1548836
SLPI, Human, pAb	Hycult Biotech	Cat#HP9024; RRID: AB_2286624
Human pIgR Antibody	R&D systems	Cat#MAB27171; RRID: AB_3657978
Monoclonal Antibody to CC16	Origene Tech	Cat#AM26360PU-N; RRID: AB_11216041
Anti-RSV Antibody, clone 133-1H	Merck	Cat#MAB8262; RRID: AB_95302
Anti-Rabbit IgG, Alexa Fluor 488	Thermo Fisher Scientific	Cat#A-11034; RRID: AB_2576217
Anti-Mouse IgG1, Alexa Fluor 647	Thermo Fisher Scientific	Cat#A-21240; RRID: AB_2535809

**Chemicals and recombinant proteins**		

DMEM	Thermo Fisher Scientific	Cat#12634-028
Opti-MEM	Thermo Fisher Scientific	Cat#31985062
BEpiCM-b	Sciencell	Cat#3211
advanced DMEM/F-12	Thermo Fisher Scientific	Cat#12634-028
B-27 Supplement, serum free	Thermo Fisher Scientific	Cat#17504001
HEPES	Thermo Fisher Scientific	Cat#15630080
GlutaMAX	Thermo Fisher Scientific	Cat#35050-061
Hydrocortisone	Sigma-Aldrich	Cat#H0888
Epinephrine hydrochloride	Sigma-Aldrich	Cat#E4642
N-acetyl-L-cysteine	Sigma-Aldrich	Cat#A9165
Nicotinamide	Sigma-Aldrich	Cat#N0636
3,3′,5-Triiodo-L-thyronine sodium salt	Sigma-Aldrich	Cat#T6397
Penicillin/streptomycin	Thermo Fisher Scientific	Cat#15070-063
Primocin	InvivoGen	Cat#ant-pm-2
Amphotericin B	Thermo Fisher Scientific	Cat#15290018
Gentamicin	Sigma-Aldrich	Cat#G1397
Vancomycin	Sigma-Aldrich	Cat#SBR00001
A83-01	Tocris	Cat#2939/10
Y-27632	Selleck Chemicals	Cat#S1049
Rapamycin	Sigma-Aldrich	Cat#553210
DAPT	Thermo Fisher Scientific	Cat#15467109
DMH1	Selleck Chemicals	Cat#S7146
TTNPB	Cayman	Cat#16144-1
RSPO3-Fc Fusion Protein CM	U-Protein Express	Cat#R001
Recombinant human Heregulin-β1	PeproTech	Cat#100-03
Recombinant human FGF10	PeproTech	Cat#100-26
Recombinant human HGF	PeproTech	Cat#100-39H
Recombinant human EGF	PeproTech	Cat#AF-100-15
Recombinant human FGF7	PeproTech	Cat#100-19
Recombinant Interleukin-1β	PeproTech	Cat#200-01
Nirsevimab	Provided by AstraZeneca	N/A
Collagen IV	Sigma-Aldrich	Cat#C7521
PureCol	Advanced BioMatrix	Cat#5005
Cultrex Basement Membrane Extract, Type 2	Trevigen	Cat#3532-010
TrypLE express enzyme	Thermo Fisher Scientific	Cat#12605010
CryoStor CS10	STEMCELL Technologies	Cat#07930
SiR-tubulin	Spirochrome AG	Cat#SC002
DAPI	Sigma-Aldrich	Cat#D9542
ProLong Gold	Thermo Fisher Scientific	Cat#P36934
VX-809	Selleck Chemicals	Cat#S1565
VX-661	Selleck Chemicals	Cat#S7059
VX-445	MedChemExpress	Cat#HY-11177
VX-770	Selleck Chemicals	Cat#S1144
Calcein green (AM)	Invitrogen	Cat #C34852
Forskolin	Sigma-Aldrich	Cat#F3917
G418 (Geneticin)	Invivogen	Cat#GNL-40-03
ELX-02 (Exaluren)	MedChemExpress	Cat#HY-114231

**Cultureware**		

96-well culture plates	Greiner Bio-One	Cat# 655182
24-well culture plates	Greiner Bio-One	Cat# 662160
12-well culture plates	Greiner Bio-One	Cat#665165
6-well culture plates	Greiner Bio-One	Cat#657160
6.5 mm Transwell with 0.4 μm Pore Polyester Membrane Insert	Corning	Cat#3470
Nunc dishes (35 mm) with UpCell Surface	Thermo Fisher Scientific	Cat#174904

**Commercial kits**		

RNeasy Mini Kit	Qiagen	Cat#74104
iScript cDNA synthesis kit	Bio-Rad	Cat#1708891
iQ SYBR Green Supermix	Bio-Rad	Cat#1708880

**Oligonucleotides**		

qPCR Primers	This papers, Table S4	N/A

**Experimental models: Primary cells and cell lines**		

Human Nasal Epithelial Cells (HNEC)	UMC Utrecht	Protocol ID: 16–586, and 21-044
Human Epithelioma-2 (HEp-2)	ATCC	Cat#CCL-23

**Experimental models: viral strains**		

mKate-RSV-A2	Hotard et al.^37^	N/A
RSV-A and -B clinical isolate strains	Langedijk et al.^38^	N/A

**Equipment**		

Thunder Imager 3D live Cell with	Leica	N/A
Leica DFC9000 GCT camera	Leica	N/A
Zeiss LSM800 confocal microscope	Zeiss	N/A
Nanodrop spectrophotometer	Thermo Fisher	N/A
CFX96 real-time detection machine	Bio-Rad	N/A
BD FACSJazz	BD Bioscience	N/A

**Software and algorithms**		

Leica LAS X Software	Leica	https://www.leicamicrosystems.com/
Zen Blue Software	Zeiss	https://www.zeiss.com/
ImageJ/FIJI	NIH, Fiji developers	https://imagej.net/Fiji/
GNU Image Manipulation Program		https://www.gimp.org/
Prism 10	GraphPad Software Inc.	https://www.graphpad.com/
R (version 4.3.3)	R Core	https://www.R-project.org/
RStudio	RStudio	http://www.rstudio.com/
BBrowserX	BioTuring	https://bioturing.com/
Talk2Data	BioTuring	https://bioturing.com/
Vinci software	BioTuring	https://bioturing.com/
Seurat (v.5.1.0)	Hao et al.^39^	https://satijalab.org/seurat/index.html
CEL-Seq2 (v 1.0)	Hashimshony et al.^40^	https://github.com/yanailab/CEL-Seq-pipeline
DESeq2 (v.1.36.0)	Love et al.^41^	https://github.com/mikelove/DESeq2
Correlescence (v.0.0.7)	Eugene Katrukha	https://github.com/ekatrukha/Correlescence

**Deposited data**		

Bulk RNA-seq de-identified raw count	NCBI Gene Expression Omnibus (GEO) database	GEO: GSE318346
Single cell RNA-seq de-identified raw count	NCBI Gene Expression Omnibus (GEO) database	GEO: GSE318347

### Experimental model and study participant details

#### Human materials and informed consent

Nasal brushings from healthy volunteers (*n* = 10 independent donors), PCD subjects (*n* = 5 independent donors), and CF subjects (*n* = 9 independent donors) were collected as previously described.^18^ All donors gave informed consent and this study was approved by the Institutional Medical Research Ethics Committee of the University Medical Center Utrecht (Toetsingscommissie Biobank Utrecht, the Netherlands). Samples were allocated to experimental groups based on predefined clinical diagnosis and genotype, and all donors were subjected to identical culture conditions and experimental treatments. Donor characteristics, including age, sex, affected gene, and genetic variants, are provided in Table S3. Due to limited sample sizes within donor groups, the study was not powered to detect sex- or age-specific effects, and no stratified analyses were performed.

#### Cell lines

Human Epithelioma-2 (HEp-2) cells were obtained from the American Type Culture Collection (ATCC). Cell line identity was varified by ATCC quality control procedures, and cells were routinely tested for mycoplasma contamination.

#### Isolation and expansion of airway epithelial basal cells from nasal brushings

Nasal brushing-derived airway epithelial basal cells were isolated essentially as previously described.^18^ In brief, dissociated single cells were seeded in a 50 μg/mL collagen IV-precoated 6-well cell culture plate and cultured in isolation medium consisting of 50% (v/v) BEpiCM-b and 44% (v/v) advanced DMEM/F-12 (Ad-DF) supplemented with 2% (v/v) B-27, 10 mM HEPES, 1% (v/v) GlutaMAX supplement, 1% (v/v) penicillin/streptomycin, 0.5 μg/mL Hydrocortisone, 1.25 mM N-Acetyl-L-cystein, 100 μg/mL Primocin, 1 μM ALK5 inhibitor A83-01, 0.5 μg/mL (±)- Epinephrine hydrochloride, 5 μM Y-27632, 2% RSPO3-Fc Fusion Protein conditioned medium, 50 nM Recombinant Human Heregulin-beta 1, 100 ng/mL Recombinant human Fibroblast growth factor 10 (FGF10), 25 ng/mL Recombinant human Hepatocyte growth factor (HGF). To prevent microbial infections, the following antibiotics were added during the first week of isolation: 250 μg/mL Amphotericin B, 50 μg/mL Gentamicin and 50 μg/mL Vancomycin. After the first week, 5 μM NOTCH inhibitor DAPT and 5 nM Rapamycin were added to the medium. Cells were cultured at 37°C with 5% CO_2_ and medium was refreshed three times a week until 80–90% confluency was reached. Cells were passaged using TrypLE express enzyme. Passage 1 cells were frozen in CryoStor CS10 supplemented with 5 μM Y-27632 to create a master cell bank and passage 2 cells were frozen to create a work cell bank. Population doublings (PD) were calculated as PD = 3.32×(log(cells harvested/cells seeded)).

#### Differentiation of HNECs in submerged and ALI cultures

For differentiation experiments in submerged cultures, basal cells (BCs; passage 3–12) were cultured on conventional culture plates which were pre-coated with 30 μg/mL PureCol. Cells were seeded in a density of 0.2–0.3 × 10^6^ cells per cm^2^ and cultured in 280 μL per cm^2^ expansion medium until reaching 100% confluency after approximately 5–7 days. Afterward, culture medium was switched to a differentiation medium consisting of 98.5% (v/v) Ad-DF with 100 nM 3,3′,5-Triiodo-L-thyronine sodium salt, 0.5 μg/mL hydrocortisone, 0.5 μg/mL (±)-Epinephrine hydrochloride, 50 nM A83-01, 100 nM retinoic acid agonist TTNPB, 0.5 ng/mL recombinant human EGF and 1% (v/v) penicillin/streptomycin, which was supplemented with 5 μM DAPT and 5 μM BMP inhibitor DMH1. Medium was refreshed three times per week. Cultures were washed with 100 μL PBS for 5 min once a week. ALI-differentiation on 24-well transwells inserts (6.5 mm with 0.4 μm Pore Polyester Membrane) was conducted as previously described,^18^ with minor changes. In short, 0.2 million BCs were seeded on transwell inserts and cultured in expansion medium until confluency was reached. Then, medium was switched to differentiation medium supplemented with 500 nM A83-01. Next, apical fluid was removed to create an air-liquid interface. After 3–5 days medium was switched to the final differentiation medium, which consisted of differentiation medium with 5 μM DAPT and 5 μM DMH1.

#### Generation of organoid from S-diff HNECs

Conversion of submerged-differentiated airway epithelia into organoids was conducted essentially as previously described with ALI-differentiated cultures.^17^^,^^18^ First, BCs were differentiated at submerged conditions on PureCol coated thermo-reactive Nunc dishes with UpCell Surface (35 mm) for at least 18 days. To detach the differentiated epithelial monolayer, the culture medium was substituted for ice-cold Ad-DF and culture dishes were placed on ice for a period of 5–10 min. Subsequently, the detached epithelial monolayer was disrupted into epithelia fragments, which were subsequently embedded in 30 μL droplets of basement membrane extract (BME). Next, solidified BME droplets were overlayed with airway organoid medium consisting of 95.5% (v/v) Ad-DF with 2% (v/v) B-27, serum free, 1% (v/v) GlutaMAX, 10 mM HEPES, 1.25 mM N-acetyl-L-cysteine, 5 mM Nicotinamide, 500 μM A83-01 and 1% (v/v) penicillin/streptomycin supplemented with DAPT (5 μM), FGF7 (5 ng/mL) and FGF10 (10 ng/mL). Epithelial fragments, self-organized into organoids (1–2 days), were subsequently transferred in 4 μL droplets of BME on a prewarmed 96-well plate. For comparative experiments, organoids were generated from ALI-cultures and used in FIS assays as previously described.^17^^,^^18^

### Method details

#### Immunofluorescent microscopy

2D differentiated cultures on transwells and on 96-well plastic tissue culture plates were fixed in 4% paraformaldehyde for 15 min, permeabilized in 0.3% (vol/vol) Triton X-100 in PBS for 30 min and treated with blocking buffer, consisting of 1% (wt/vol) BSA, and 0.3% (vol/vol) Triton X- in PBS for 60 min. Primary antibodies (1:500 in blocking buffer) were incubated for two hours. Afterward, cells were washed three times with PBS and incubated with secondary antibodies (1:500 in blocking buffer) and DAPI (1:1000) for 30 min in the dark. After three washings with PBS, cultures differentiated on plastic were stored in 150 μL PBS at 4°C and cultures differentiated on transwells were cut from the plastic insert and mounted with ProLong Gold antifade reagent without DAPI on slides. 3D airway organoids were stained as previously described.^17^ Images were acquired with a Leica THUNDER imager using 5, 10, 20×, and 40× dry objectives, and processed using Leica software. Stained surface signal of β-tubulin IV and MUC5AC was quantified using GNU Image Manipulation Program (GIMP; https://www.gimp.org/) by using the color threshold and mask function.

#### RNA isolation, quantitative real-time PCR and bulk RNA-sequencing

Total RNA was extracted using the RNeasy kit according to the manufacturer’s protocol. RNA yield was measured with a Nanodrop spectrophotometer. cDNA was generated using the iScript cDNA synthesis kit according to the manufacturer’s protocol. Quantitative real-time (qPCR) was performed using iQ SYBR Green Supermix and a CFX96 real-time detection machine, and primers described in Table S4. Gene expression was calculated using the comparative 2-ΔΔCT method and normalized against the housekeeping genes *ATP5B*, *GAPDH*, and *YWHAZ*. For bulk RNA sequencing, extraction and library preparation followed an adapted version of the CELseq2 protocol.^40^ Sequencing was performed with the Illumina NextSeq (Sequencing depth: STANDARD (10M reads/sample)). Sequencing results were mapped to the human genome (hg38) using R software. Bulk RNA-seq count normalization and differential gene expression were analyzed using the DESeq2 package.^41^ Significantly differentially expressed genes of different sample groups were selected using a log2 fold change ((Padj<0.01 and |log2 Fold change|>1) and adjusted using the Bayesian shrinkage (sh_log2FC).

#### Single cell RNA sequencing analysis

HNECs differentiated in 6-well plastic plates (*n* = 3 independent healthy donors) were dissociated from the culture plate using TrypLE express enzyme and resuspended to a single cell suspension. Samples were pelleted, washed with PBS, resuspended in FACS buffer (PBS0, 1% FBS, 0.5 mM EDTA and DAPI) and strained (35 μm). 376 cells per donor were immediately sorted into 384-well cell-capture plates containing ERCC spike-ins (Agilent), RT primers and dNTP (Promega) using a BD FACSJazz. ScRNA-seq was performed according to the SORT-seq protocol.^20^ In short, cells were lysed for 5 min at 65°C, RT and second-strand mixes were dispensed by the Nanodrop II liquid handling platform (GC Biotech) and double-stranded cDNAs of single-cell was pooled and transcribed following the CEL-seq2.^40^ Samples were sequenced with 75.000 reads per cell on a Illumina NextSeq. For the analysis of the scRNA-seq data, paired-end reads were aligned to the human transcriptome using Burrows-Wheeler Alignment tool.^42^ Read 1 was used to assign reads to map reads to the correct cells and read 2 was mapped to gene models. Only uniquely mapped reads were used for further analysis. Reads duplicated were removed by excluding reads with identical library, cellular and molecular barcodes. Transcript counts were adjusted to the number of expected molecules based on counts, possible UMI’s and Poissonian counting statistics. In total, 181 cells had to be excluded during quality control measures, leaving 335 cells for donor 1, 284 cells for donor 2 and 328 cells for donor 3. Clustering and analysis of the sequencing results were performed using the Seurat pipeline^39^ and BioTuring Software. To extract cell type–specific gene signatures, the Human Lung Atlas^24^ was queried using the Talk2Data module in BioTuring. Enriched gene expression profiles for basal, secretory, and ciliated epithelial cells were retrieved based on annotated *ex vivo* nasal epithelial subsets. These gene sets were exported and used for AUCell scoring^43^ in the BBrowserX module of BioTuring to quantify gene signature enrichment across clusters within S-diff HNEC cultures. Visualization of single-cell data was performed using Vinci software of BioTuring.

#### High speed video microscopy and ciliary beat frequency analysis

Submerged-differentiated HNEC, cultured in plastic 96-well plates and ALI cultures in 24-well transwell cultures, were imaged on a Thunder Imager 3D live Cell with a DFC9000 GCT camera in a 37°C heated chamber. The presence of cilia in the analyzed videos was confirmed by a life-cilia staining 200 nM SiR-tubulin, which was incubated for 4 h prior to imaging. Cultures were washed twice with PBS prior to imaging and acquisition took place within 30 min after washing. Videos were taken with a 40× objective at three randomly picked locations per culture with an imaging speed of at least 200 frames per second (fps). The ciliary beat frequency was estimated using “Temporal ICS”command of Correlescence v.0.0.6 plugin for ImageJ.^44^ The full version of the corresponding code is available online (https://github.com/ekatrukha/Correlescence), but in short, it consists of the following steps. At the initial stage, the average intensity image was subtracted from recorded time-lapse to remove a static component. Then for each pixel position of the time image stack we calculated normalized autocorrelation function over different time delays. The period of pixel’s intensity oscillations was estimated as a position of a first maximum of the autocorrelation function with a tolerance above 0.2. The frequency was calculated as the period’s reciprocal value. As an output, we obtained an output image of the same X,Y dimensions with its “intensity” values equal to the frequency. All frequencies below 0.1 Hz and above 28 Hz were excluded. To filter for background noise we removed pixel clusters smaller than 25 pixels. For figures and statistics we used the mean CBF value per video.

#### Forskolin-induced swelling (FIS) assay

CFTR function and CFTR modulator responses were determined in organoids in a forskolin-induced swelling (FIS) assay as previously described.^18^ CF airway organoids were pre-treated with CFTR correctors: 5 μM VX-809, 5 μM VX-661, 5 μM VX-445, or vehicle control for 48 h. CFTR-dependent organoid swelling was measured after stimulation with 5 μM forskolin and the CFTR potentiators 5 μM VX-770.

#### RSV infections in submerged-differentiated airway epithelia

For the infection experiments we used mKate-RSV-A2^37^ for the optimization procedures and RSV-A and -B clinical isolate strains obtained by the INFORM study.^38^ RSV viral strains were propagated in HEp-2 cells as previously described.^10^ Briefly, HEp-2 cells were seeded at 2 × 10^6^ cells in a T25 flask one day prior to infection or until 90% of a confluent monolayer was obtained. When confluent, cells were infected with RSV (multiplicity of infection (MOI) = 0.1) and incubated for 3–5 days till 60% of cytopathic effects was observed. Virus stocks were snap frozen on dry ice and stored at −80°C until further use. Viral titer was calculated according to 50% tissue culture infectious dose (TCID50). S-diff HNECs cultured in a 96-wells format and differentiated for 28 days were infected in duplicates. Cell cultures were initially washed once with Dulbecco’s modified Eagle’s medium (DMEM) at 37°C, with 5% CO_2_ for 10 min and subsequently, washing medium was removed and cells were incubated with viral inoculums diluted in 1:1 DMEM:Opti-MEM medium at 37°C, with 5% CO_2_ for 2 h. When experiments involved treatment application, virus was incubated with Nirsevimab prior to cell incubation for 1 h. After infection was carried out, inoculum was aspirated and cells were rinsed twice with PBS and left in culture for the defined period of infection upon addition of differentiated medium. Cells were refreshed every two days and negative controls consisted of mock infections, where medium alone was used without virus. To investigate RSV growth kinetics, S-diff HNECs and HEp-2 cells were cultured on 24-wells plate and infected with RSV at a MOI of 0.01 or 3. After 2h of incubation (37°C, 5% CO_2_), viral inoculum was collected for titration by TCID50 to verify virus input and mentioned as the zero hours timepoint for growth curves. Subsequently, cells were rinsed twice with PBS and differentiation medium was added. For the multi-step growth kinetic assay, at 2, 24, 48 and 72 h post-infection (HPI), infected cells were scraped in medium and freeze-thawed to release cell-bound virus. For the single-step growth kinetic assay, virus was harvested at 2, 6, 24, 32 and 48 HPI. Viral supernatants of different timepoints were collected and titrated by TCDI50 in HEp-2 cells.

#### Cell-based RSV detection ELISA

An indirect enzyme-linked immunosorbent assay (ELISA) was used to quantify RSV F -protein. Cells were fixated prior to staining with 80% acetone in PBS (v/v) at 4°C for 15–30 min. RSV F-protein was detected using a mouse anti-RSV antibody used 1:5000 in casein, which was incubated at 37°C, 5% CO_2_ for 1 h. After incubation, cells were washed 4 times with 0.1% PBS/Tween 20 (PBS/T) and subsequentially, horseradish peroxidase (HRP)-conjugated goat anti-mouse diluted 1:2000 in PBS was incubated at 37°C, 5% CO_2_ for 1 h. After a 6-times final washing step with 0.1% PBS/T, 100 μL/well of substrate solution tetramethylbenzidine (TMB) was added followed by incubation in the dark at room temperature for 7 min. Hereafter, 50 μL/well of stop solution (2N H2SO4) was added and RSV specific F-protein levels were quantified by measuring the optical density (OD) per well at 450 nm. Uninfected controls were included on each plate to correct for background signal. The ELISA was validated in the presence of mKate-RSV-A2; MOI = 2 or the absence of infection (mock condition), resulting in maximum (max) signal values and minimum (min) signal values. Viral infection was quantified as previously described by measuring OD signal at 450 nm. CV values were calculated according to the following formula: % CV = (sd of means)/(mean of means) × 100. Max and min infectivity enabled Z′-factor calculation of each 96-well plate according to the following formula: Z′-factor = 1 − (3× (σp + σn)/(μp − μn)), where σp is the standard deviation of the max signal wells (*n* = 36 per plate, mkate RSV; MOI = 1), σn is the standard deviation of the min signal wells (*n* = 36 per plate, mock condition), μp is the mean of the max signal wells and μn is the mean of the min signal wells. Three biological replicates were performed.

### Quantification and statistical analysis

GraphPad Prism 9.3.0 was used to perform statistical analyses. Statistical tests and significance are indicated in figure legends.

## Supplemental information

Document S1. Figures S1–S9 and Tables S3 and S4

Table S1. Enriched genes of cell type-specific clusters in submerged-differentiated cultures based on scRNA-seq, related to Figure 2 (A) Enriched genes of basal cell cluster. (B) Enriched genes of secretory cell cluster. (C) Enriched genes of ciliated cell cluster.

Table S2. Differentially expressed genes in submerged-differentiated cultures, ALI-cultures, and basal cells based on bulk RNA-seq, related to Figure 3 (A) S-diff vs. BCs. (B) ALI-diff vs. BCs. (C) S-diff vs. ALI-diff.

Document S2. Article plus supplemental information
