# Supplementary material for: Small molecule-directed differentiation of submerged-cultured human nasal airway epithelia for respiratory disease modeling
Source: Cell Rep Med. 2026 Mar 23;7(4):102692. doi: 10.1016/j.xcrm.2026.102692 (PMC13130688; doi:10.1016/j.xcrm.2026.102692)
Supplement: Document S1. Figures S1–S9 and Tables S3 and S4 [file mmc1.pdf]

Cell Reports Medicine, Volume *7*

Supplemental information

Small molecule-directed differentiation of submerged-cultured human nasal airway epithelia for respiratory disease modeling

Henriette H.M. Dreyer, Georgia-Nefeli Ithakisiou, Sacha Spelier, Malina K. Iwanski, Eugene Katrukha, Jonne Terstappen, Lisa W. Rodenburg, Aditi Shekhar, Loes A. den Hertog-Oosterhoff, Shannon M.A. Smits, Isabelle S. van der Windt, Lotte T. Azink, Linda H.M. Bijlard, Koen Passier, Sam F.B. van Beuningen, Robert Jan Lebbink, Eric G. Haarman, Cornelis K. van der Ent, Lukas C. Kapitein, Louis J. Bont, Jeffrey M. Beekman, and Gimano D. Amatngalim

Supplemental figures

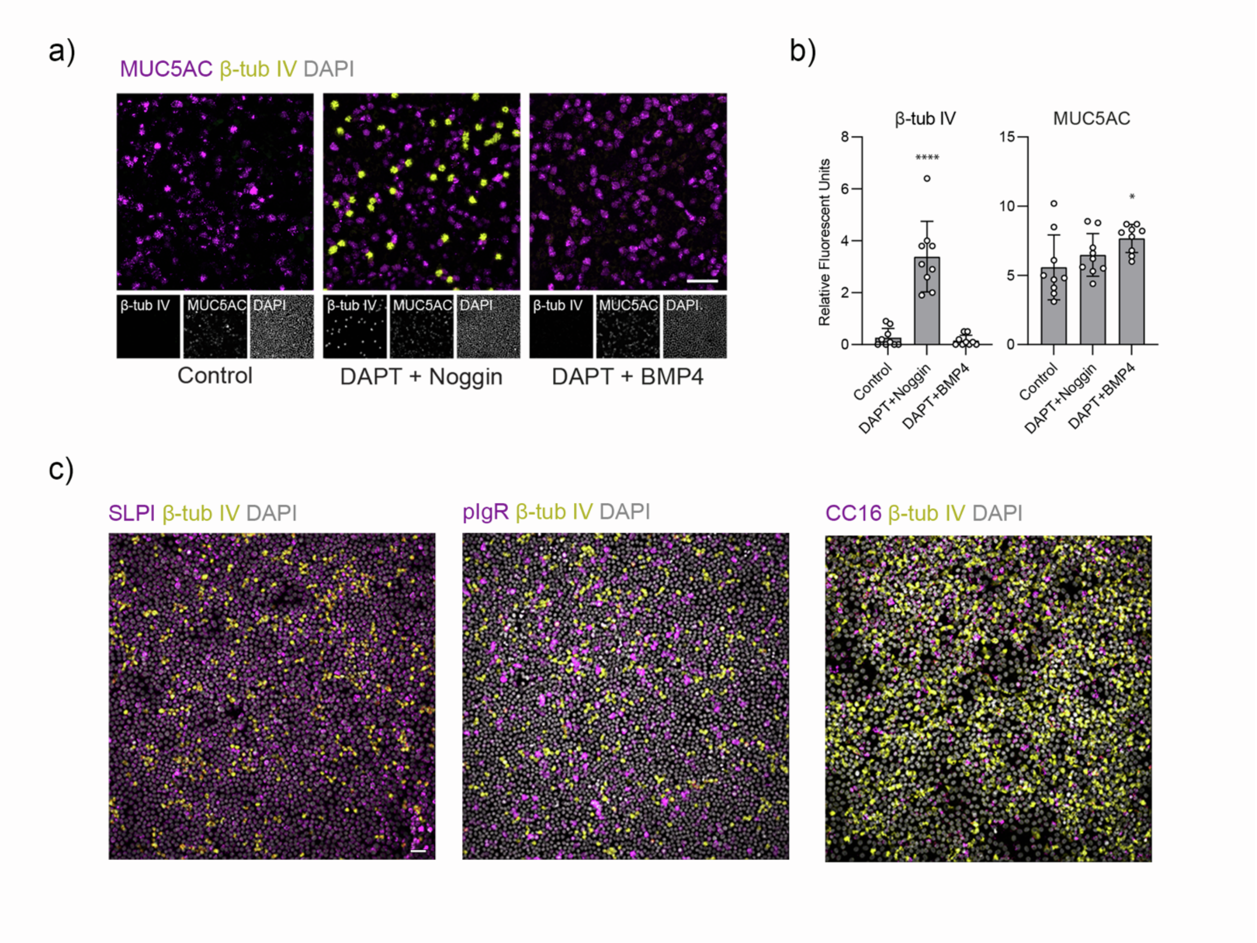

Figure S1: The effect of BMP on submerged differentiation and secretory cell characterization. Related to Figure 1. a) Representative immunofluorescent images of a HNEC differentiated submerged with and without the Notch inhibitor DAPT, the BMP inhibitor Noggin, or recombinant BMP4 for 18 days. Cells were stained for the secretory cell marker MUC5AC (purple), ciliated cell marker β-tubulin IV (β-tub IV; yellow) and DAPI. b) Quantification of β-tubulin IV (β-tub IV) and MUC5AC signal (n=3 images for 3 independent donors). c) Representative immunofluorescent images of S-diff HNEC stained for the secretory cell markers SLPI, pIgR, and CC10 (purple), together with tubulin IV (β-tub IV; yellow). Scale bars: 50 µm. Data are presented as mean ± SD with individual data point. Statistical significance was tested using a two-way ANOVA with Dunnett's multiple comparison test: ****: p<0.0001.

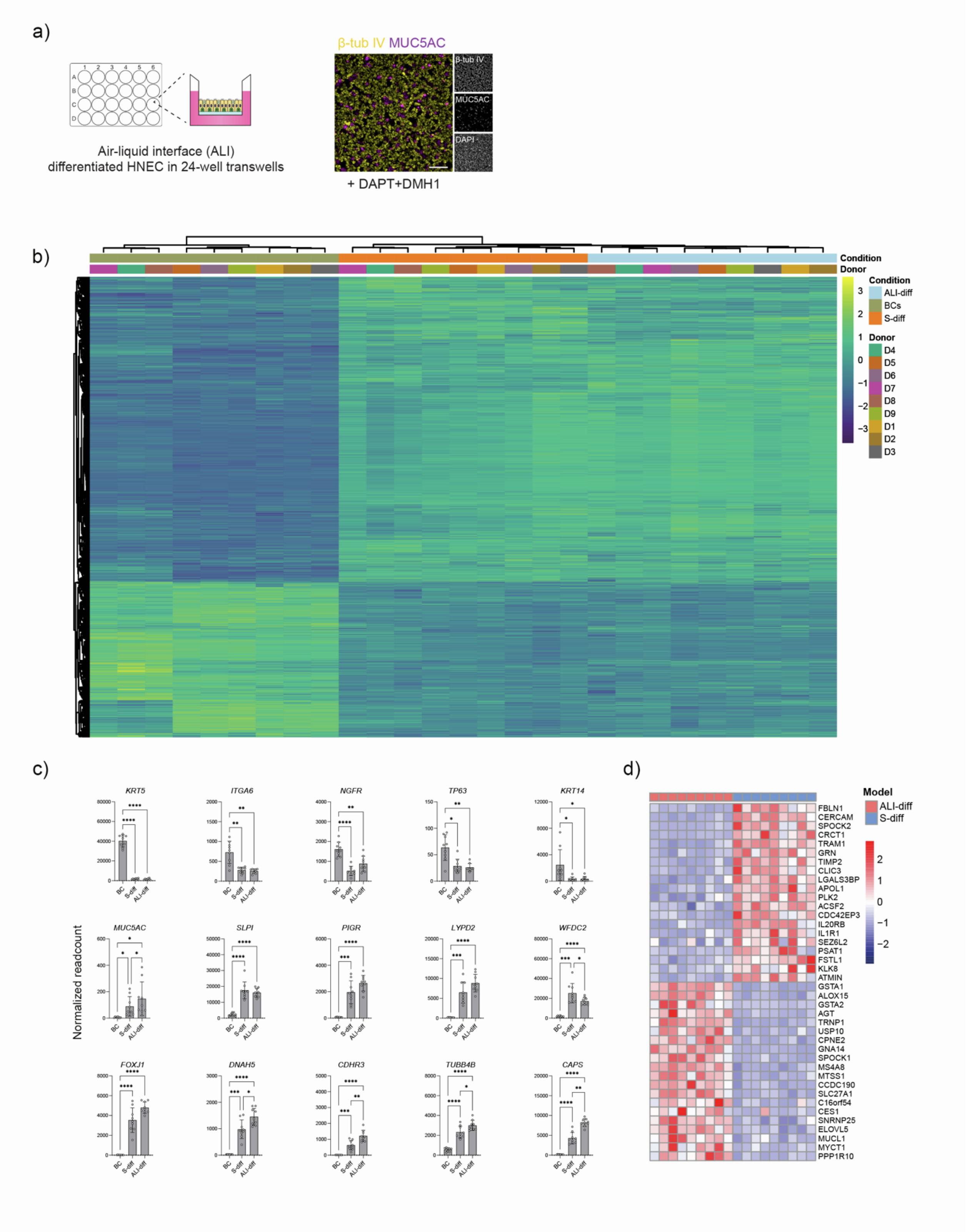

Figure S2: Bulk RNAseq comparison between S-diff HNEC, BCs, and ALI-diff HNEC. Related to Figure 3. a) Representative immunofluorescent images of ALI-cultures differentiated with DAPT and DMH-1 for 18 days. Cells

were stained for the secretory cell marker MUC5AC (purple), ciliated-cell marker β-tubulin IV (β-tub; yellow) and DAPI. Scale bar: 50 µm. b) Heatmap showing expression of all DEGs in submerged-differentiated HNEC compared to basal cells (BCs) and ALI-differentiated cultures (n=9 independent donors). c) Normalized mRNA read counts of a selection of epithelial markers in BCs, S-diff, and ALI-differentiated HNEC, including the basal cell markers: *KRT5*, *ITGA6, NGFR, TP63*, and *KRT14*; Secretory cell markers: *MUC5AC*, *SLPI, PIGR, LYPD2,* and *WFDC2*; Ciliated cell markers: *FOXJ1, DNAH5, CDHR3, TUBB4B,* and *CAPS*. d) Heatmap showing marker gene expression of top 20 enhanced and reduced DEGs in S-diff HNEC compared to ALI-HNEC (n=9 independent donors). Data are presented as mean ± SD, and individual data point. Statistical significance was tested using a two-way ANOVA with Dunnett's multiple comparison test *: $p<0.05$, **: $p<0.01$, ***: $p<0.001$, ****: $p<0.0001$.

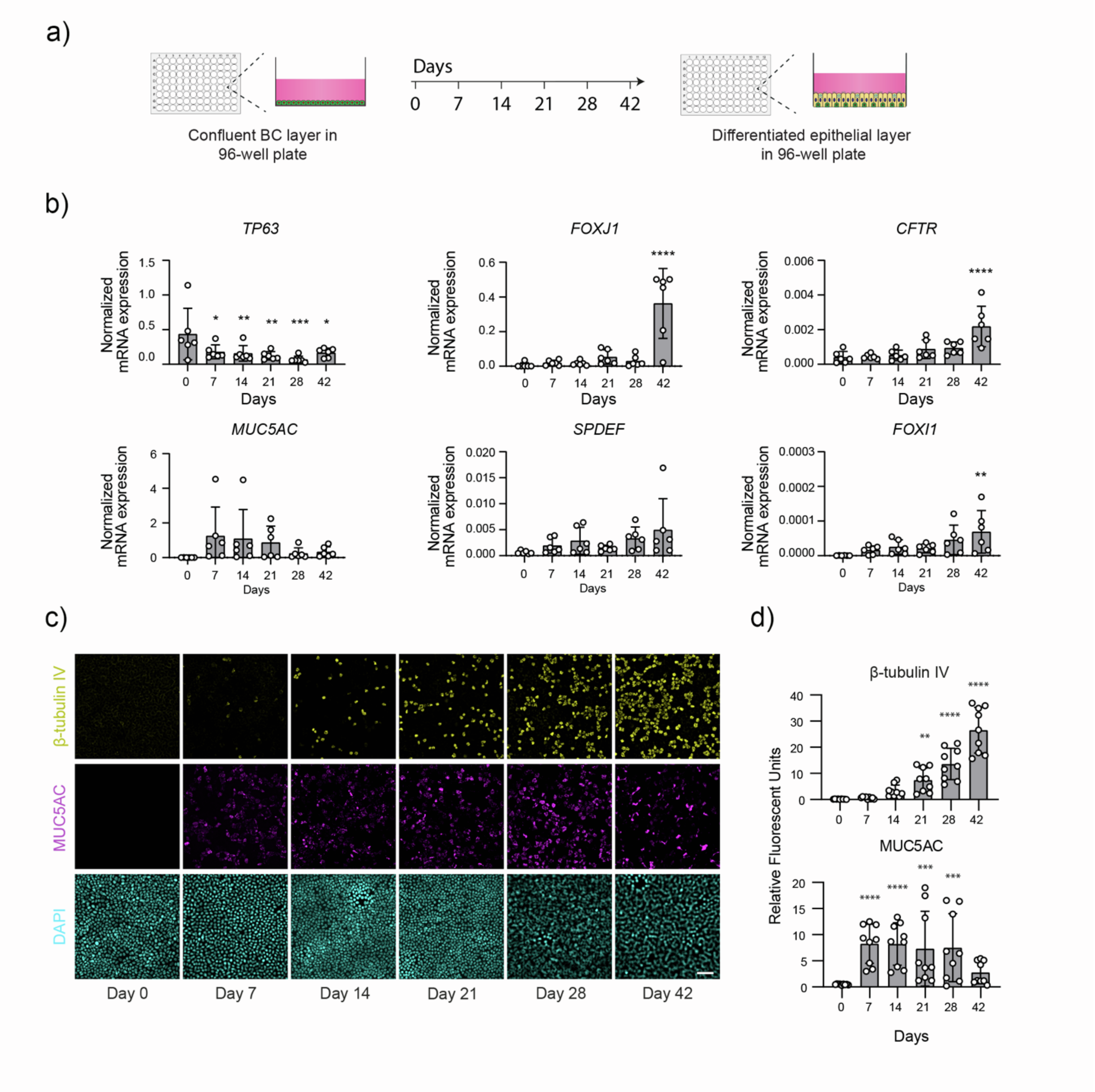

Figure S3: Changes in cell composition during submerged differentiation. Related to Figure 3. a) Graphic illustration showing the time course experiment set-up. Submerged cultures were used for experiments at six time points between day 0 and day 42 of differentiation. b) Quantitative PCR comparing the expression of *TP63, MUC5AC*, *SPDEF, FOXJ1, CFTR* and *FOXI1* of submerged cultures differentiated for 0, 7, 14, 21, 28, and 42 days (n=2 replicates for 3 independent donors). mRNA expression was normalized to the average expression of housekeeping genes. Statistical significance was tested using a two-way ANOVA with Dunnett's multiple comparison test compared to day 0. c) Representative immunofluorescent images of submerged cultures

differentiated for 0, 7, 14, 21, 28, and 42 days. Cells were stained for the secretory cell marker MUC5AC (purple), ciliated cell marker β-tubulin IV (yellow), and DAPI (cyan). Scale bar: 50 μm. d) Quantification of β-tubulin IV and MUC5AC (n= 3 independent donors and n=3 different locations). Data are presented as mean ± SD with individual data point. Statistical significance was tested using a two-way ANOVA with Dunnett's multiple comparison test to day 0. Only significant differences are shown. *: $p<0.05$, **: $p<0.01$, ***: $p<0.001$, ****: $p<0.0001$.

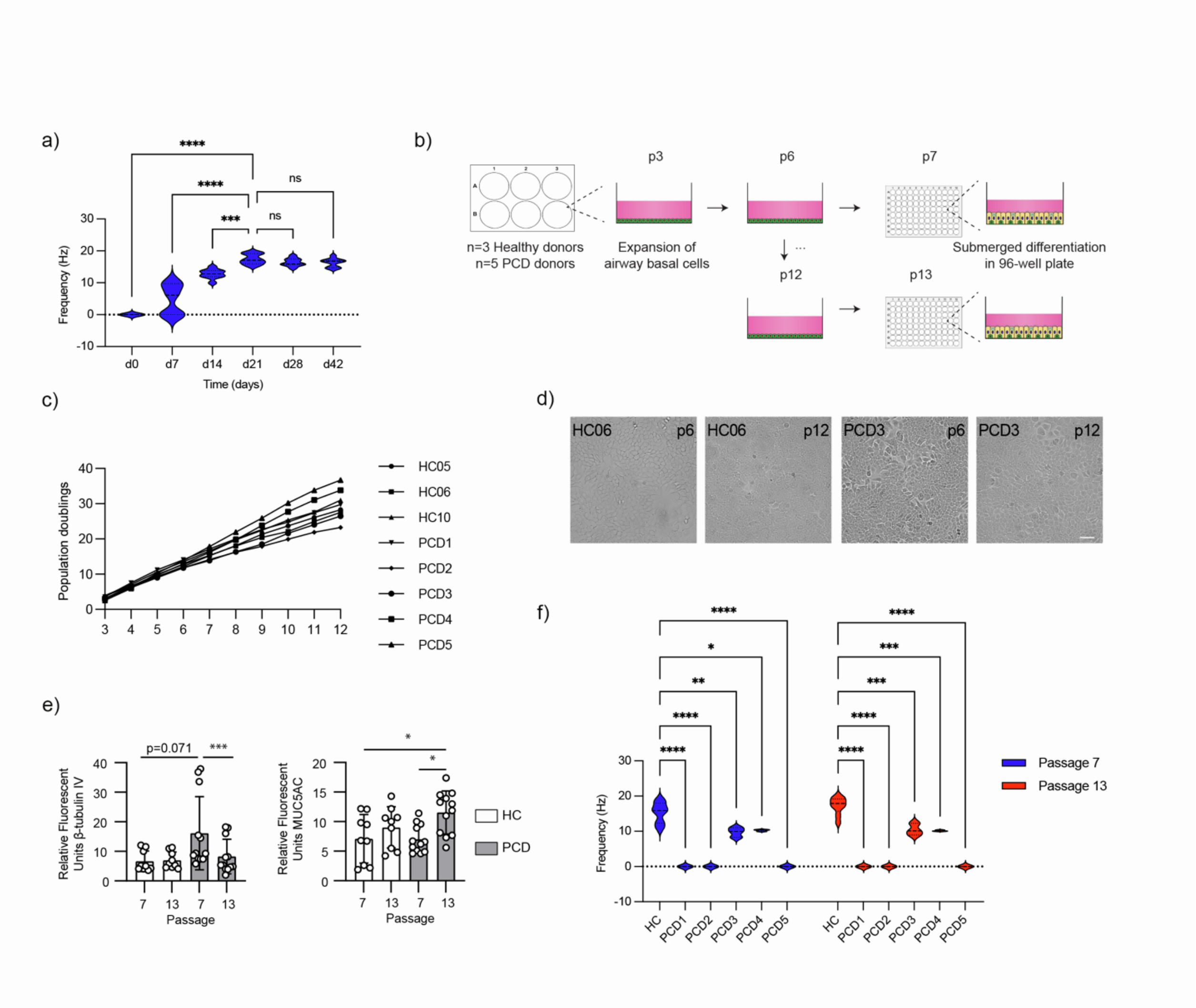

Figure S4: Long-term expansion and submerged differentiation of S-diff HNEC. Related to Figure 4. a) Ciliary beat frequency (CBF, in Hz) of submerged-differentiated cultures measured at day 0, 7, 14, 21, 28, and 42 (n=3 independent healthy donors). Data are shown as violin plots. b) Graphic illustration showing the long-term expansion of BCs in 6-well plates and differentiation in 96-well culture plates at passage seven (p7) and p13. c) Population doublings (PD) measured in BC cultures from donors HC (5,6,10) and PCD1-5. PD=3.32×(log(cells harvested/cells seeded)). d) Representative brightfield images of BC cultures from a HC and PCD subject at p6 and p12. Images were taken before passaging. Scale bar: 50 µm. e) Quantification of β-tubulin IV and MUC5AC signal measured in p7 and p13 S-diff HNEC of HC (n=3 independent subject) and PCD subjects (n=4 independent subjects). Data are presented as mean ± SD, and individual datapoints. f) CBF in Hz of submerged-differentiated HC (n=3) and PCD (n=5) donor cultures at p7 and p13, shown as violin plots. Statistical significance was tested using (a) a Dunnett's

multiple comparison test to day 21. (e,f) a Tukey's multiple comparison test. ns = non-significant, *: $p<0.05$, ***: $p<0.001$, ****: $p<0.0001$.

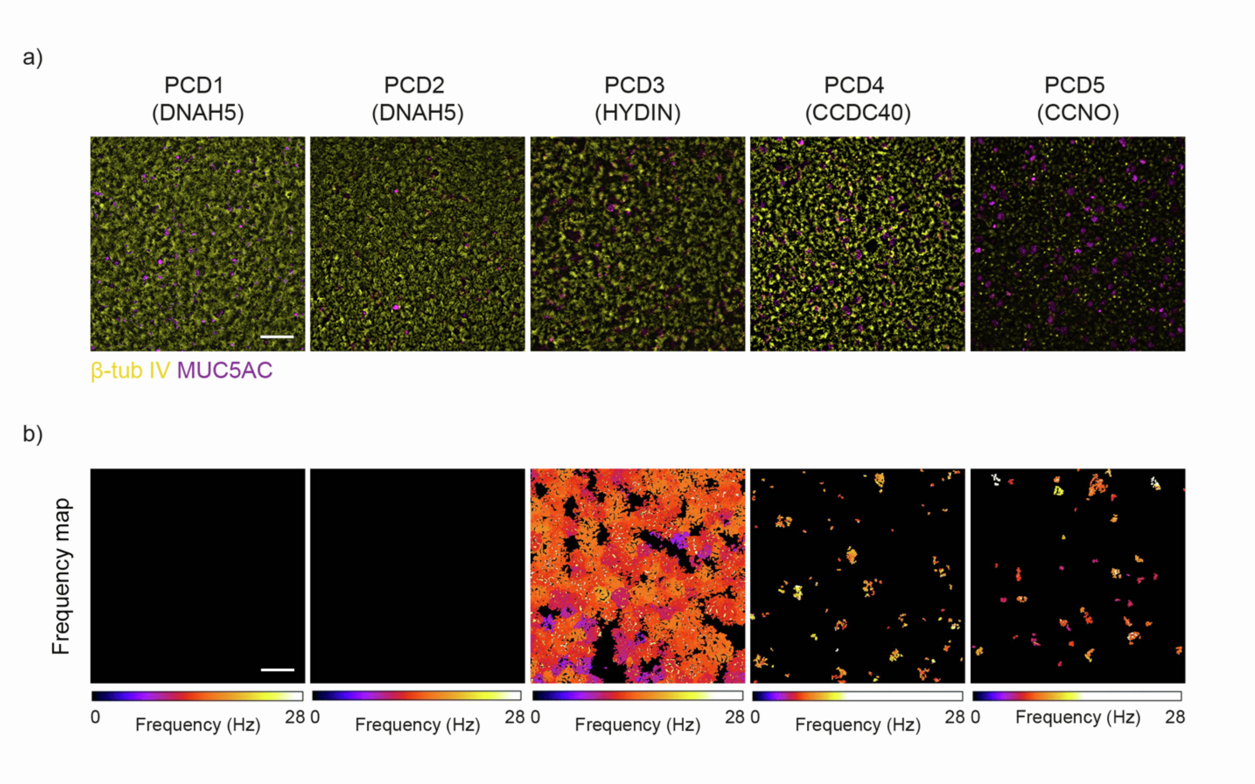

Figure S5: Differentiation and ciliary function in ALI-differentiated nasal cultures of PCD donors. Related to Figure 4. a) Representative immunofluorescence images of ALI-differentiated nasal epithelial cultures from PCD donors (PCD1–5), differentiated for 18 days in the presence of DAPT and DMH-1. Cultures were stained for the secretory cell marker MUC5AC (purple), ciliated cell marker β-tubulin IV (yellow), and DAPI (blue). b) Representative CBF maps of ALI-differentiated PCD cultures. Scale bars: 50 µm. Quantified CBF data are shown in Figure 4d.

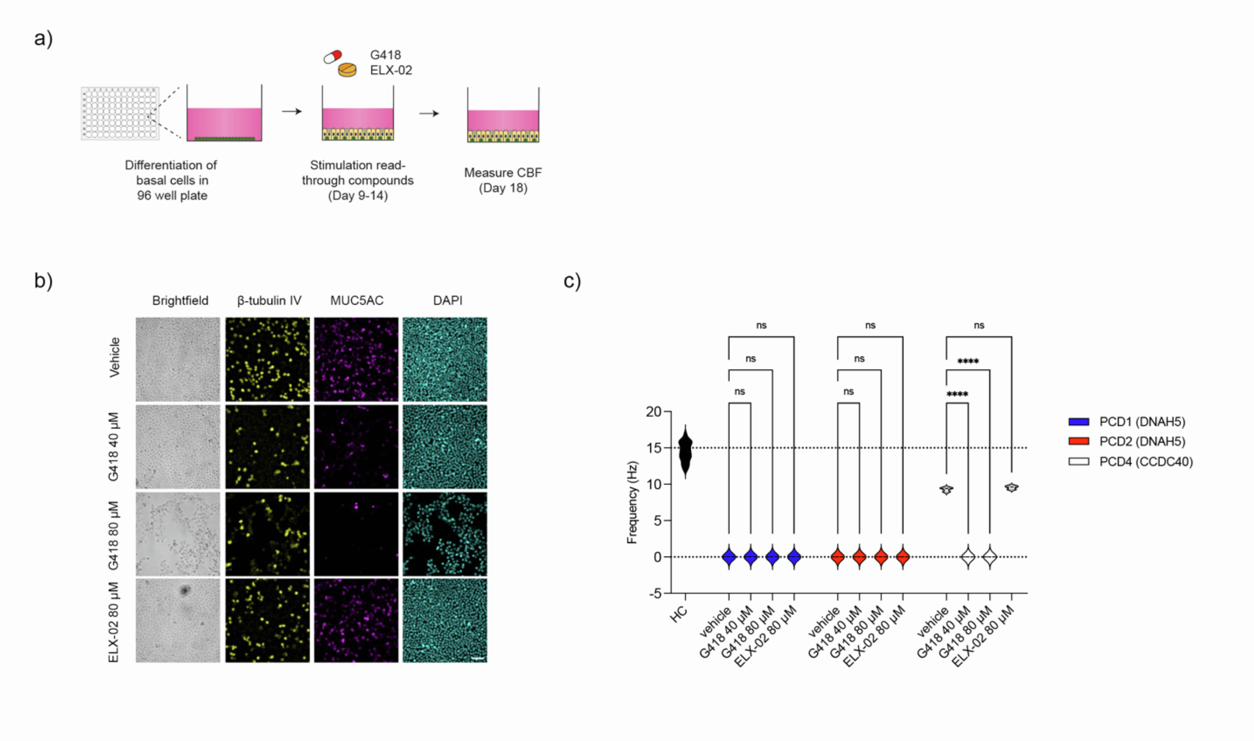

Figure S6: Quantification of CBF in submerged-differentiated cultures of PCD donors with PTC mutations in response to readthrough compounds. Related to Figure 4. a) Schematic overview of the experimental setup for readthrough compound treatment. Compounds were added between day 9 and day 14 of differentiation, followed by CBF measurements on day 18. b) Representative immunofluorescence images of submerged-differentiated HNEC from a PCD donor, treated with vehicle, G418 (40 µM and 80 µM), or ELX-02 (80 µM). Cultures were stained for the secretory cell marker MUC5AC (purple), ciliated cell marker β-tubulin IV (yellow), and DAPI (blue). G418 showed signs of cytotoxicity at higher concentration. Scale bar: 50 µm. c) Ciliary beat frequency (CBF, in Hz) in submerged-differentiated HNEC from PCD donors with PTC mutations in DNAH5 (PCD1, PCD2) or CCDC40 (PCD4), treated with G418 or ELX-02. No improvement in CBF was observed. In CCDC40-mutant cultures, CBF was reduced after treatment, consistent with cytotoxicity. Experiments were repeated twice per donor. Data are shown as violin plots; dotted lines indicate average CBF of HC cultures. Statistical significance was tested using one-way ANOVA with multiple comparison test. ns = non-significant; ****: $p<0.0001$.

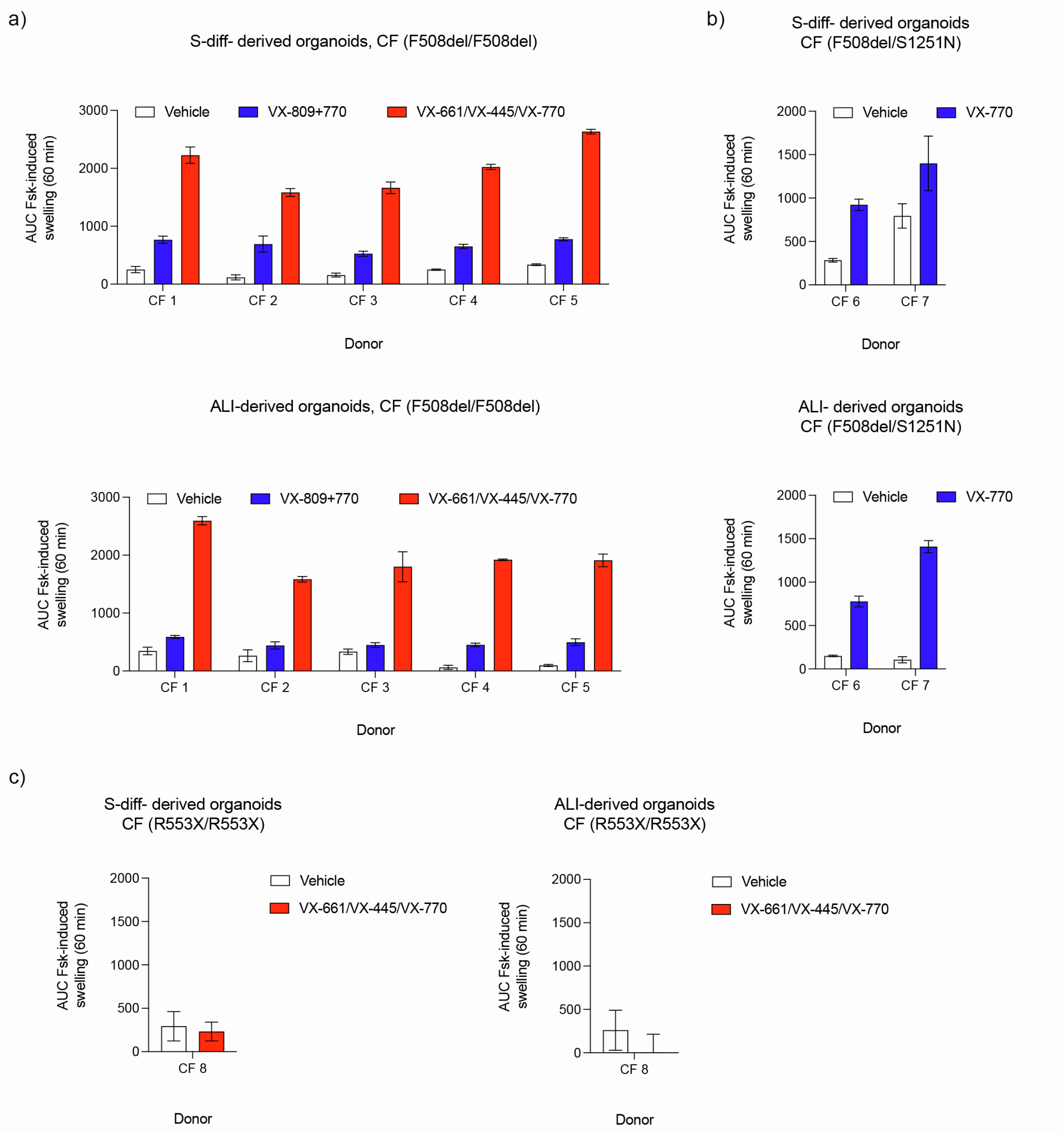

Figure S7: CFTR modulator response validation in airway organoids derived from ALI- and S-diff cultures. Related to Figure 5. a) CFTR modulator responses measured in airway organoids generated from S-diff and ALI-differentiated HNEC of CF donors with F508del/F508del genotype (n=5 independent donors). Organoids were pre-treated for 48 hours with vehicle, VX-809, or VX-661/VX-445, followed by forskolin-induced swelling (FIS) assays with acute stimulation using forskolin (Fsk), VX-770, or vehicle. b) FIS measurements in organoids derived from S-

diff and ALI-diff HNEC of CF donors with F508del/S1251N genotype (n=2 independent donors), following acute stimulation with the CFTR potentiator VX-770. c) FIS responses in organoids derived from S-diff and ALI-diff HNEC of a CF donor with R553X/R553X genotype. Organoids were pre-treated with vehicle or VX-661/VX-445 for 48 hours, followed by acute stimulation with Fsk, VX-770, or vehicle. FIS results are presented as area-under-the-curve (AUC) plots, calculated from the percentage change in organoid surface area relative to t = 0 (normalized area), measured at 15-minute intervals over 60 minutes. Data are presented as mean ± SD. FIS values were used in the correlation analysis shown in Figure 5j.

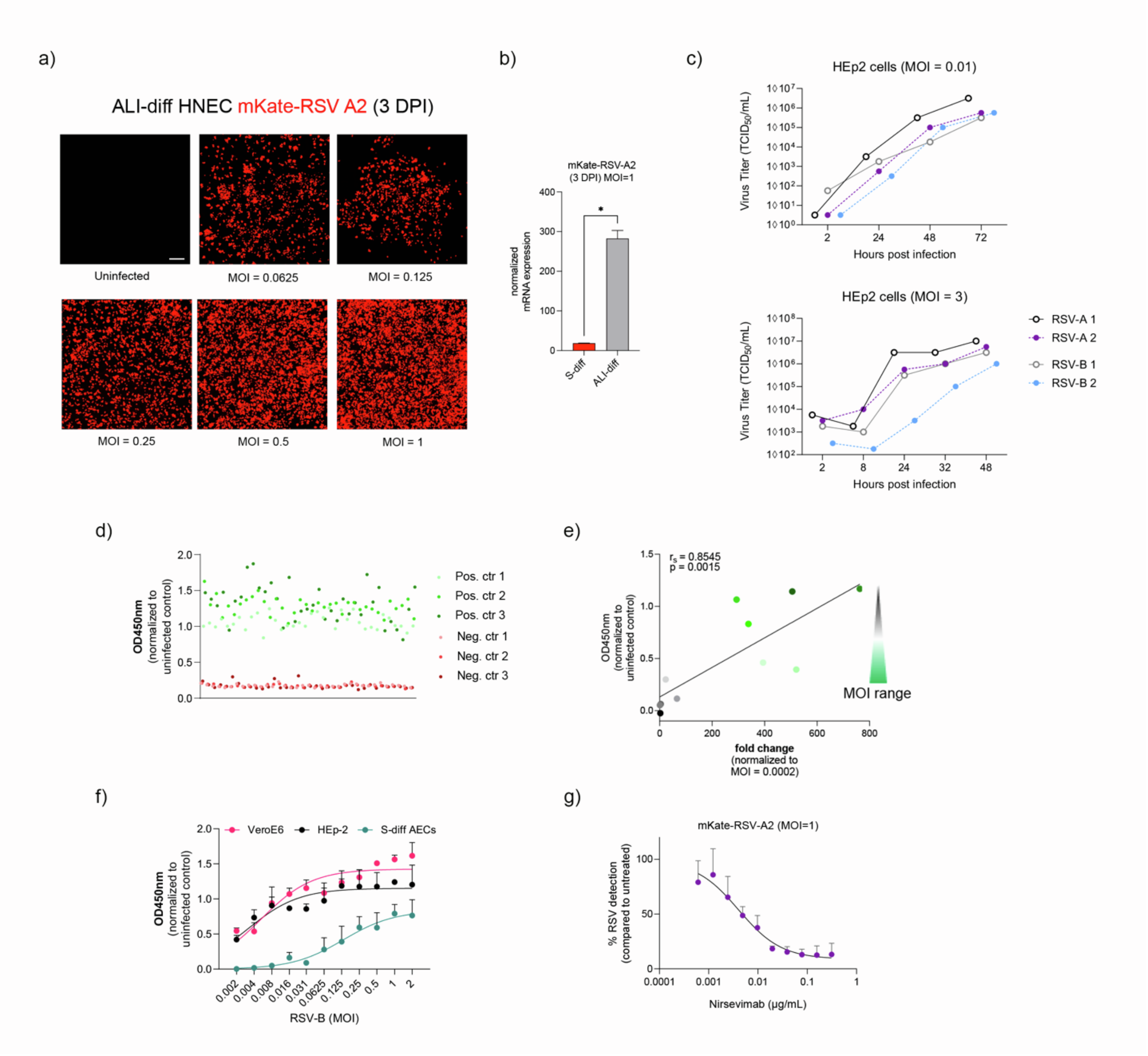

Figure S8: Validation experiments of RSV infections. Related to Figure 6. a) Live imaging of mKate-RSV-A2 infections with different MOIs of ALI-differentiated HNEC at 3 DPI. Scale bar: 50 µm. b) Quantification of RSV mRNA expression by qPCR in S-diff and ALI-differentiated HNEC infected with RSV-A2 mKate (MOI = 1) at 3 DPI, normalized to housekeeping genes. c) HEp-2 cells were infected with RSV-A2 mKate at MOI = 0.01 or 3. Virus growth kinetics was determined by titrating supernatant and cell-bound RSV at different time intervals following infection. d) Replicate experiments of three 96-wells plates to assess the reliability and robustness of the ELISA assay in which n=36 wells were infected with RSV-A2 mKate at an MOI of 2 at 3 DPI (=max OD signal) and in which n=36 wells S-diff HNEC remained uninfected (=min OD signal). CV values were calculated according to the following formula: % CV = (sd of means)/ (mean of means) × 100. Z'-factor of each 96-wells plate was

calculated according to the following formula: Z'-factor = $1 - (3\times(\sigma p + \sigma n)/(\mu p - \mu n))$, where σp is the standard deviation of the max signal wells (n=36 per plate, RSV-A2 mKate; MOI = 1), σn is the standard deviation of the min signal wells (n=36 per plate, mock condition), μp is the mean of the max signal wells and μn is the mean of the min signal wells. e) Correlation between qPCR (fold change) and ELISA (OD450nm). f) Cell-based ELISA with S-diff HNEC, VERO E6, and HEp-2 cells, infected with different MOIs of a RSV-B clinical isolates. g) Assessment of the neutralization activity of Nirsevimab against mKate-RSV-A2. Data are presented as mean ± SD, from two individual experiments with two technical replicates per condition in each experiment; SD is indicated by error bars.

a)

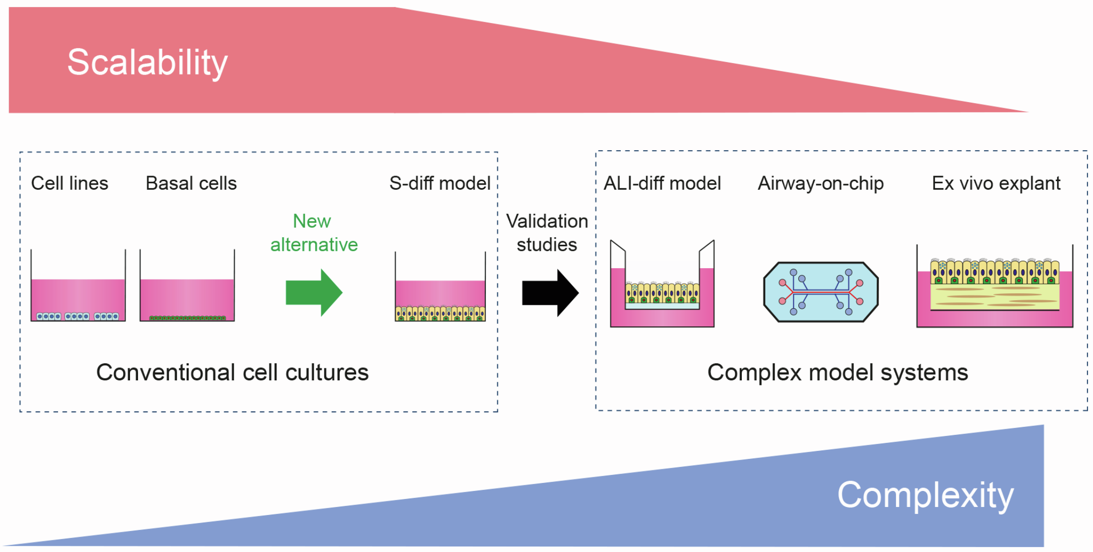

b)

Model	HEp-2 cells	S-diff HNEC	ALI-diff HNEC
Time to usable culture	4 days	21-42 days	21-28 days
Estimated cost (96 wells)	~ € 18,00	~ € 76,80	~ € 468,50
Setup complexity	Easy	Moderate	High
Donor-specific biology	None	Present	Present
Epithelial complexity	Minimal	Moderate	High
Throughput potential	High	Medium-High	Low

Figure S9: Positioning and comparison of S-diff cultures within airway epithelial model systems. a) Schematic workflow illustrating the positioning of S-diff cultures in experimental pipelines. S-diff cultures can serve as a scalable alternative to undifferentiated basal cells and transformed cell lines and can be used in screening assays prior to validation in more complex model systems such as ALI-differentiated cultures. b) Comparative overview of HEp-2 cells, S-diff, and ALI-diff HNEC cultures. The table summarizes key differences in time, cost for generating

96-well cultures, setup complexity, donor-specific biology, epithelial complexity, and throughput potential. S-diff cultures provide a balance between biological relevance and experimental feasibility.

Table S3: Donor Characteristics

Donor ID	Age	Sex	Status	Gene	Variant 1	Variant 2
HC1	32	Male	HC			
HC2	25	Male	HC			
HC3	27	Female	HC			
HC4	25	Female	HC			
HC5	33	Male	HC			
HC6	35	Male	HC			
HC7	20	Female	HC			
HC8	21	Female	HC			
HC9	29	Female	HC			
HC10	21	Female	HC			
PCD1	23	Male	PCD	*DNAH5*	Q3462*	Y2790fs
PCD2	31	Female	PCD	*DNAH5*	c.13338+5G>A	Q3462*
PCD3	27	Male	PCD	*HYDIN*	R2786*	G4623*
PCD4	15	Male	PCD	*CCDC40*	A83fs	Q619*
PCD5	59	Male	PCD	*CCNO*	V265fs	V265fs
CF1	30	Female	CF	*CFTR*	F508del	F508del
CF2	33	Male	CF	*CFTR*	F508del	F508del
CF3	26	Male	CF	*CFTR*	F508del	F508del
CF4	41	Female	CF	*CFTR*	F508del	F508del
CF5	20	Male	CF	*CFTR*	F508del	F508del
CF6	22	Female	CF	*CFTR*	F508del	S1251N
CF7	32	Female	CF	*CFTR*	F508del	S1251N
CF8	13	Male	CF	*CFTR*	F508del	S1251N
CF9	22	Female	CF	*CFTR*	R553*	R553*

* indicates a premature stop codon; fs indicates a frameshift mutation.

Table S4: Primer sequences

Gene	forward sequence (5' to 3')	reverse sequence (5' to 3')
TP63	CCACCTGGACGTATTCC ACTG	TCGAATCAAATGACTAGGAG GGG
MUC5AC	ATTTTTTCCCCACTCCT GATG	AAGACAACCCACTCCCAACC)
FOXJ1	GGAGGGGACGTAAATC CCTA	TTGGTCCCAGTAGTTCCAGC
SPDEF	ATGAAAGAGCGGACTT CACCT	CTGGTCGAGGCACAGTAGTG
FOXI1	CCGACAACTTCCCCTTC TAC	CCAAGGAGGCTGTGCTAGAG
CFTR	CAACATCTAGTGAGCA GTCAGG	CCCAGGTAAGGGATGTATTGT G
ATP5B	TCACCCAGGCTGGTTCA GA	AGTGGCCAGGGTAGGCTGAT
GAPDH	TGCACCACCAACTGCTT AGC	GGCATGGACTGTGGTCATGA G)
YWHAZ	CTGGAACGGTGAAGGT GACA	AAGGGACTTCCTGTAACAATG CA
RSV-A N gene	CATCCAGCAAATACAC CATCCA	TTCTGCACATCATAATTAGGA GTATCAA
